# Molecular nanotechnologies of gelatin-immobilization using macrocyclic metal chelates

**DOI:** 10.3402/nano.v5.21485

**Published:** 2014-02-07

**Authors:** Oleg V. Mikhailov

**Affiliations:** Department of Analytical Chemistry, Kazan National Research Technological University, Kazan, Russia

**Keywords:** nanotechnology, nanoparticles, coordination compounds, self-assembly, macrocyclic metal chelate

## Abstract

This article is a review of recent developments in the self-assembled nanostructures based on chelate coordination compounds. Molecular nanotechnologies of self-assembly of 3*d*-element aza- and thiazametalmacrocyclic complexes that happen in nanoreactors on the basis of metal hexacyanoferrate(II) gelatin-immobilized matrix under their contact with water solutions containing various (N,O,S)-donor atomic ligands and organic compounds having one or two carbonyl groups have been considered in this review. It has been noted that the assortment of macrocyclic metal chelates obtained as a result of using molecular nanotechnologies in such specific conditions considerably differs from the assortment of metal chelates formed at the conditions traditional for chemical synthesis.

In the extensive world of modern nanotechnologies, so-called molecular nanotechnologies rank high. Their basic conceptual idea is the creation of a particular target object according to the ‘upwards’ principle in which it is ‘composed’ of separate nanoscale blocks or separate molecules The major chemical reactions used nowadays in the aforementioned nanotechnology include the processes of the so-called self-assembly of the molecules, some of which have already been discussed in the literature, for example (1–4). In some cases, an association of molecules of the initial substances gets a specific orientation under the influence of a particular ‘template’; now the processes of such self-assembly are widespread in the synthesis of macrocyclic compounds, and, first of all, the so-called metal macrocyclic compounds, the structure of which contains atoms of different *d-* and *f-*elements (5–12). The role of a mold in the synthesis of these objects is played by the metal ion, which then enters the composition of the metal macrocyclic compound formed during self-assembly. As a rule, each of them presents a metal complex with a chelate polydentate ligand obtained not according to the scheme metal ion+ligand → complex classical for the metal complexes but to the scheme metal ion+‘building blocks’ of the future ligand, so-called ligand synthons or ligsons → complex. In particular, it is necessary to emphasize that the metal ion present in the reaction system – the so-called template – does not simply ‘conduct’ the process of self-assembly; in its absence, this process does not occur at all. Rather often, the processes of self-assembly play a key role in the synthesis of such macroheterocyclic compounds which do not contain metal atoms in their structures: in this case, metal chelates formed in the beginning are subjected to demetallation. In this respect, the above reactions currently take the dominating position in the synthesis of aza-, azaoxo-, and thiazamacrocycles; crown-ethers; and other systems with closed contours containing various heteroatoms in their ‘skeletons’. The final products of these reactions, which are called template synthesis in the chemistry of macrocyclic compounds, which have a set of non-trivial physical and chemical properties, are applied widely; the list of fields where they are used includes metallurgy and medicine, industrial biotechnology and catalysis, microelectronics and agriculture, and numerous other fields of human activity.

Currently, the processes of self-assembly of metal macrocyclic compounds are practically always implemented in conditions ‘traditional’ for chemistry, namely, in the solution, in the solid phase, and, sometimes, in the gas phase. Traditionally, chemical synthesis is performed in macro- and, much less often, microreactors. However, so that the synthetic modes develop and improve, it is of interest and important to introduce a *nano*component not only in the chemical reaction with the purpose of obtaining metal macrocycles and performed, as was already mentioned, according to the ‘upwards’ principle but also in the medium where this reaction proceeds. Simply speaking, this is the idea of performing the above process of self-assembly in the nanoscale reactors. This can be done by using specific but, in general, quite accessible to chemists, objects such as biopolymer-immobilized organizing systems on the basis of polypeptides or polysaccharides. These systems contain intermolecular cavities large enough in concepts of the ‘nanoworld’ which can be considered original molecular *nano*reactors and in which the various chemical processes in general and those of self-assembly in particular can be implemented in principle. As a result, the *nano*-sized particles of metal macrocyclic chelates arising in the course of such processes, can be formed in the system indicated above. This paper is concerned with these systems.

## 1. GIM as an organizing medium in the processes of self-assembly of metal macrocycles

Gelatin-immobilized matrices (GIM) with gelatin as the polymeric binding agent belong to the biopolymeric systems which, in principle, can be used to implement the processes of self-assembly of metal complexes. This high-molecular compound easily forms the so-called gels (solutions in the low-molecular liquids that have some solid-state properties, in particular, the absence of fluidity at low transverse strain, the ability to keep their form, and considerable strength and elasticity). It has been well-known for a long time (see 13–19) that, by its chemical nature, gelatin is a polydisperse mixture of low-molecular peptides with the general formula **I** (R_1_, R_i_, R_j_, R_k_ are various radicals)


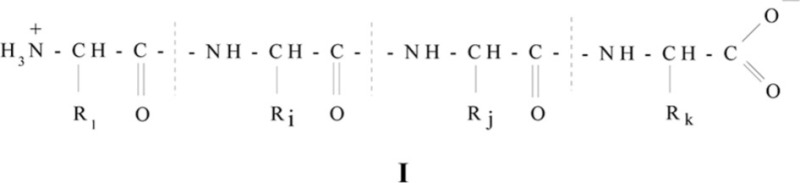


with the molecular mass *M=*50.000–70.000 or their aggregates with *M=*200.000–300.000 composed of 18 natural amino acids from a total of 20, except for cystine and cysteine. Gelatin is obtained from the natural fibrillary fiber of the protein collagen. Its role is to form the mesh structure keeping the strain in any connecting tissue – skin, bone, cartilage, sinews, and so on. Most of these amino acid sections, namely a little more than a third of the total number, is the ‘residue’ of the elementary amino acid, glycine; the second in prevalence is the ‘residue’ of proline; and the third, with a somewhat lower content, is alanine. Groups with labile sulfur and disulfide bridges are not characteristic for the structure of gelatin and collagen (13). It was established by the electron-microscopic method that the diameter of the gelatin macromolecules is 14 nm, while their length is 2,850 nm (16). These numbers are in good agreement with the similar data found in ref. (17) on the measurements of the light dispersion and viscosity of gelatinous solutions. These data show that the gelatin molecule is strongly asymmetric and anisometric (16, 17). Each molecule consists of three parallel α-chains with almost identical *M* values of 95.000 so that its molecular mass is approximately 280.000–290.000. However, for now, there are no exhaustive data on the distribution of gelatin macromolecules in the biopolymeric bulk.

The structure and properties of collagen and gelatin were repeatedly studied over the last several decades (see, in particular, 16–40). Molecules of these high-molecular compounds consist of three polypeptide chains with practically identical molecular weights, two of which – the so-called α1-chains – are usually practically identical with respect to the set and sequence of amino acids, while the third, the so-called α2-chain, in this respect differs from the two others (16, 17, 27). The typical stoichiometric composition of collagen expressed in the number and variety of α-chains in its macromolecule is (α1)_2_α2; less often it is (α1)_3_ (37). At the transformation of collagen into gelatin, the polydisperse mixture containing single (α1 and α2), double (β_11_ and β_12_), and triple (γ) polypeptide chains, which, unlike collagen macromolecules, are formed as balls or as clots. The structure for the gelatin molecules in which the *left*-handed spiral structure is attributed to each of such α-chains is proposed (24, 25, 27). In the given structure, all polypeptide spirals are weaved with each other in a unique *right*-handed spiral; the hydrogen bonds play the largest role in its stabilization (24). The α2-chains are characterized by the same set of the polypeptide fragments as the α1-chain, but in their amino acid sequence there is less proline, hydroxyproline, and lysine; however, tyrosine, valine, leucine, histidine, and hydroxylysine prevail (19). Currently in the α1-chain, the sequence of more than 700 amino acid residues is completely established (18, 19, 32).

The high content of proline and hydroxyproline, as well as the interesting fact that, according to the chemical analysis data on the sequence of the arrangement of the amino acid residues, each third residue is glycine allowed authors (21, 37) to make two alternative assumptions concerning the geometry of the (α1)_2_α_2_, (α1)_3_ structures of the gelatin molecules, in each of which the formation of the above triple helices is postulated. However, in the structure proposed in ref. (24), one bond is needed for each amino acid residue, whereas in the structure of ref. (40), twice as many bonds are required. The unambiguous choice between these structures was impossible even after X-ray analysis, and the question about the exact gelatin structure is still under discussion. For each of the peptide fragments of the gelatin molecule, the interface of the π-electrons of the C, N, and O atoms is characteristic; therefore, all –C–C–NH–C– groups get a quasi-planar structure. The interatomic carbon–nitrogen distance in this structural fragment is 132 pm, which is much less than the length of the single C–N bond (147 pm), so its bond order is rather close to two. Having at one's disposal a polymer with such a structure, it is possible to obtain, in principle, immobilized systems with a homogeneous enough distribution of the immobilized substances in a particular part of a polymeric bulk and with good steric availability of molecules of this substance for the implementation of various chemical processes. Comparatively large intervals between the chains of the spatial mesh in the molecular structure of gelatin allow the molecules and ions of the low-molecular substances, unlike large colloid particles or macromolecules, to diffuse into the intermolecular **GIM** voids practically as easy as into the liquid phase solvents. Thus, in addition, **GIM** (both thin- and thick-layer ones) are highly transparent and plastic, which makes them very convenient to be studied by various spectroscopic methods. It is also important that the gelatin bulk is destroyed easily enough under the influence of various proteolytic enzymes, f.e. trypsin, *Bacillus mesentericus*, *Bacillus subtilis*, and so on. Therefore, the chemical compounds immobilized in it can be easily isolated from it in the form of solid phases and analyzed by the same modern physical and chemical methods as those used to study solid substances isolated from the solid or gas phase reaction systems.

The so-called template synthesis (7–10), which allows one, in principle, to obtain macrocyclic and supramolecular compounds of any complexity from rather simple fragments (the so-called ligand synthons or ligsons) play an important role in the synthetic methods of modern molecular nanotechnology called self-assembly. In the above version of self-assembly, a particular metal ion with a certain electron structure as a kind of ‘pattern’ which is sometimes called a template center or simple template, provides the formation of metal complexes from the corresponding ligsons. These metal complexes have rather specific ligands (so-called chelants), the synthesis of which in the absence of the template is either complicated or cannot be performed in general. It is easy to notice from the most general considerations that the self-assembly of metal macrocyclic compounds is always accompanied by a decrease in the general entropy Δ*S* of the reaction system, sometimes it is rather large, because their composition is much more complicated when compared with the composition of the initial substances participating in its formation. According to the classical expression for the isobaric process, namely Δ*G=*Δ*H* ^*0*^ – *T*Δ*S* ^*0*^ where Δ*G*, Δ*H* ^*0*^, and Δ*S* ^*0*^ are the change of the free energy, standard enthalpy, and standard entropy during the reaction, respectively, and *T* is temperature in K, the probability of implementing any process accompanied by the decrease in the entropy decreases with the increase in the temperature. At rather low temperatures, the processes of self-assembly due to rather large activation energy values proceed with very low velocities. At rather large temperatures, when their velocities could be practically acceptable, they are thermodynamically forbidden. The situation is somewhat rescued by imposing high pressure on the reaction system at a high temperature, but even then it takes a rather long period of time – up to several hours) (7, 9). One of the topical problems of the modern synthetic coordination and supramolecular chemistry is to ‘soften’ the conditions of template synthesis and, first of all, to provide its implementation in the so-called standard conditions, namely at *T=*298 K and *P=*101,325 Pa.

One of the possible approaches to solving this problem could be the preliminary ordering of the reaction system, that is, the ‘compulsory’ decrease of its entropy. It is easy to notice that this leads to a decrease in the slope of the linear dependence Δ*G*(*T*). In fact, since the entropy is an additive quantity, in the presence of such preliminary ordering, the relationship Δ*S* ^*0*^ *=(*Δ*S* ^*0*^ *)’+*Δ*S*_*os*_, where Δ*S* ^*0*^ is the change of the standard entropy of the reaction in the absence of the above ordering, *(*Δ*S* ^*0*^ *)’* is the change of the standard entropy of the reaction at the presence of the latter, and Δ*S*_*os*_ is the actual change of the entropy during the given ordering, holds. As a result, the expression (**2**.1) for Δ*G*’(*T*) in the system where the ‘compulsory’ decrease of the entropy takes place becomes

**Fig. 1:**
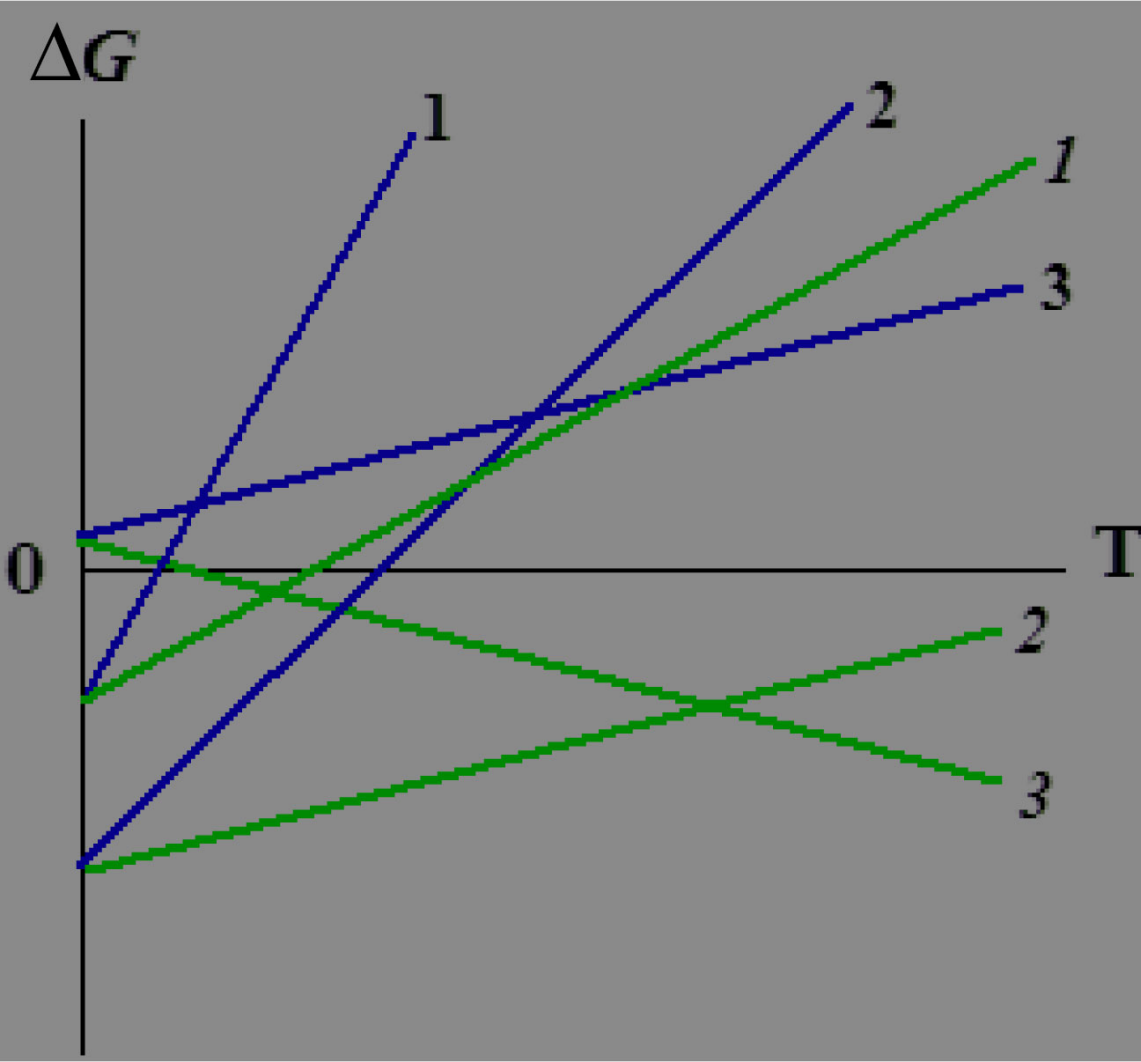
Schematic view of the Δ*G*(*T*) (**1, 2, 3**) and Δ*G*’(*T*) (***1***, ***2***, ***3***) dependences for three variants of the template process: **1**, ***1*** and **2**, ***2*** for the template processes with Δ*H<*0 which, in principle, can be implemented in the systems without a compulsory decrease in the entropy; **3**, ***3*** for the template processes with Δ*H>*0, which can be implemented only in the systems with a compulsory decrease in the entropy. The lower slope of the ***1***, ***2***, and ***3*** lines is clearly seen, unlike that of the **1, 2**, and **3** lines.

2.1 $$\Delta G\prime (T)=\Delta H^{0}-T(\Delta S^{0})\prime =\Delta H^{0}-T(\Delta S^{0}-\Delta S_{\mathrm{os}}^{0})$$

and, since $\Delta S_{\mathrm{os}}^{0}$ is negative, Δ*S* ^*0*^<0, then ∣ Δ*S* ^*0*^ – $\Delta S_{\mathrm{os}}^{0}$∣<*∣* Δ*S* ^*0*^ ∣. Accordingly, the range of the temperature values in which the given process of self-assembly is thermodynamically allowed (see Fig. 1) increases as well.

GIM implants on the basis of hexacyanoferrates(II) of various *p*-, *d*-, and *f*- elements (in further MHF **GIM**) with the nanoscale organization of the immobilized substance belong to systems with a preliminary decrease in entropy. As is known, the overwhelming majority of the current processes of self-assembly of the metal macrocycles refer to the so-called Schiff condensation, with the intramolecular formation of water due to the mobile hydrogen atoms of one ligson and oxygen atoms of another ligson (7, 9); therefore, the more mobile the hydrogen atoms in the corresponding ligson are, the higher the probability of the self-assembly in the given process is. In turn, this is directly connected with its proton donor ability. Since the macromolecules of gelatin as ampholyte and polyelectrolyte in the alkaline medium get a negative charge, the proton donor ability of compounds immobilized in it is much larger than that in the water solution; hence, the self-assembly according to the Schiff condensation in MHF **GIM** should proceed more effectively than in the solution or the solid phase. In as early as by the end of the previous century, in the works (41, 42), it has been noted that the character of the template synthesis in MHF **GIM** in a series of the triple metal ion–ligson A–ligson B systems considerably differs from that for the template synthesis in the so-called ‘traditional’ conditions, that is, in solutions and solid phase. These differences are most vividly manifested in the case of the so-called ambidentate ligsons A containing three or more donor centers and depending on the complex formation conditions, capable of being variously coordinated to a metal ion. (N,S)-donor organic compounds such as hydrazinomethanethioamide (thiosemicarbazide) H_2_N–NH–C(S)–NH_2_, hydrazinomethanethiohydrazide (thiocarbohydrazide) H_2_N–NH–C(S)–NH–NH_2_, ethanedithioamide (dithiooxamide) H_2_N–C(S)–C(S)–NH_2_, propanedithioamide-1,3 (dithiomalonamide) H_2_N–C(S)–CH_2_–C(S)–NH_2_, and so on, which can to be coordinated to a metal ion via N and S atoms, belong to such ligsons. One can use compounds both with one carbonyl group [f.e., methanal (formaldehyde) CH_2_O, propanone (acetone) H_3_C–C(O)–CH_3_] and two C=O groups [in particular, butandione-2,3 (diacetyl) H_3_C–C(O)–C(O)–CH_3_ and pentadione-2,4 (acetyl- acetone) H_3_C–C(O)–CH_2_–C(O)–CH_3_] as accompanying ligsons B providing ‘stitching’ of the metal cycles formed by the ligson A into a unique closed contour with formation of aza- and azathiamacrocyclic metal complexes. Further on, we will discuss the processes of self-assembly in this sense.

In the conclusion of this chapter, let us note two important aspects of the terminological character.

*First*. As we mentioned above, the metal ion in the template synthesis plays the role of kind of an ‘organizing and directing force’ in the formation of the metal macrocyclic compounds which is only possible or prevails in the reaction conditions from the corresponding initial organic molecules, the synthesis of which from the given initial substances in other conditions is either complicated or cannot be performed at all. The participation of a metal ion (template) in this specific quality is obligatory. If any macrocyclic ligand can be formed from simpler organic compounds without contact with the ion of the given metal, even if it is ‘composed’ of these compounds during the complex formation, the proceeding chemical reactions *do not refer* to the reactions of the template synthesis or self-assembly of metal macrocyclic compounds (7, 9). Thus, the process of self-assembly among Ni(II), salicylaldehyde, and ammonia proceeding according to the general scheme (**2**.2)


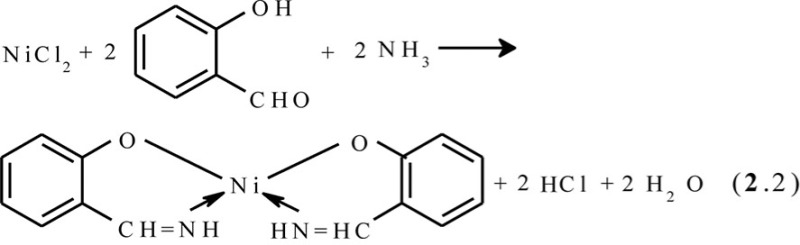


is template synthesis or self-assembly, because the ligand that is formed, salicylaldimine, does not arise at the interaction of salicylic aldehyde with NH_3_, whereas the reaction between Ni(II), salicyl-aldehyde, and any alkylamines NH_2_R according to the scheme (**2**.3)


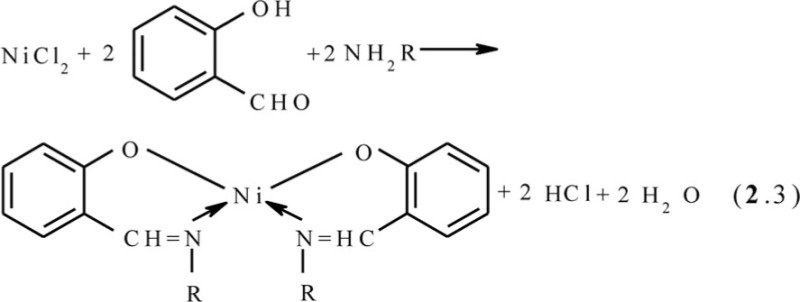


is not, because the ligand that is formed results from the direct interaction of salicylaldehyde and the corresponding alkylamine according to the general equation (**2**.4)


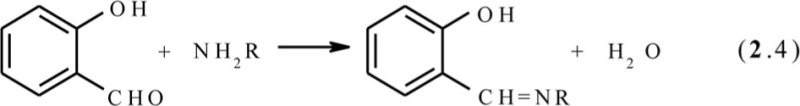


regardless of the presence of metal ion in the reaction system.

*Second*. As a result of self-assembly, as a rule, we have the formation of tetradentate chelate ligand (and it is observed in all self-assembly processes in ***GIM*** described in the literature). In that case, two types of metal chelates – macrotricyclic with three metal cycles and macrotetracyclic with four metal cycles, are theoretically possible. In this connection, it is worth entering special conventional signs of polycyclic metal complexes within the framework of analogous classification, namely to indicate for them, by means of figures in brackets, the number of atoms in metal cycles containing in these complexes. The number of these figures (three or four) will show the total number of metal cycles in this complex. The cycle numbering will be started from the leftmost one and then move clockwise along the cyclic loop perimeter. In addition, metal cycles formed as a result of ‘cross-linking’, will be indicated on the second position in the case of macrotricyclic complexes and on the second and fourth positions in the case of macrotetracyclic complexes. For example, complex having structural formula **I** will be marked as 555, complex having structural formula **II**, as (**565**), complex having structural formula **III**, as (**5656**), and so on (M–3*d*-element atom).


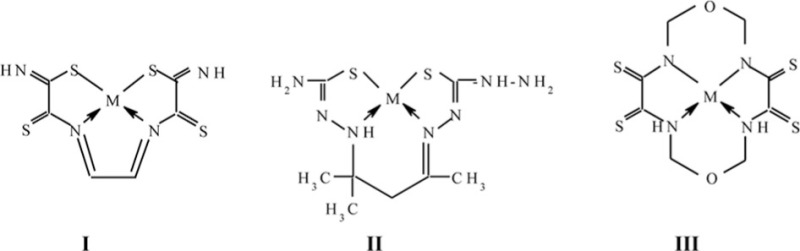


Original experimental data concerning self-assembly of macrotricyclic and macrotetracyclic 3d-element metal chelates in the specific conditions of various MHF **GIM** are presented in publications (41–92) and will be discussed below.

## 2. Self-assembly of aza- and azathiamacrocyclic metal chelates with the participation of (N,S)-ligsones and monocarbonyl compounds

The first experimentally observed case of self-assembly in MHF **GIM** is the template process at the contact of Ni_2_[Fe(CN)_6_] **GIM** with a water–alkaline (pH 12) solution containing dithiooxamide and formaldehyde leading to the formation of the diamagnetic brown (**565**)macrotricyclic compound Ni(II) with 2,8-dithio-3,7-diaza-5-oxanonandithioamide-1,9 [Ni(**L1**)] (41). On the basis of the chemical analysis data, mathematically treating the kinetic curves, UV–VIS, and IR spectroscopy in this and later works (41–45), it has been shown that in this case the process (**3**.1) takes place.


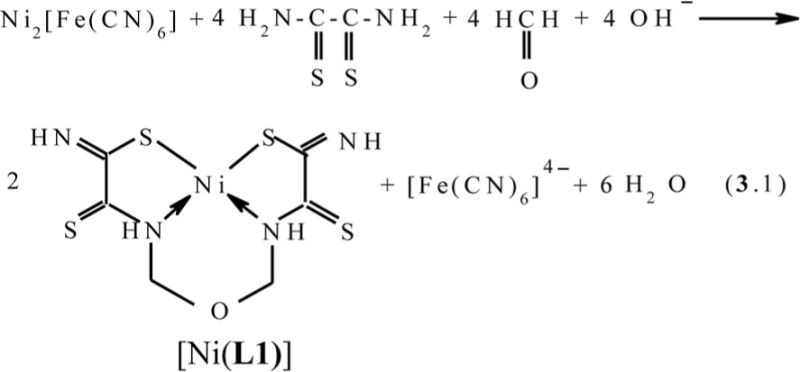


**Fig. 2:**
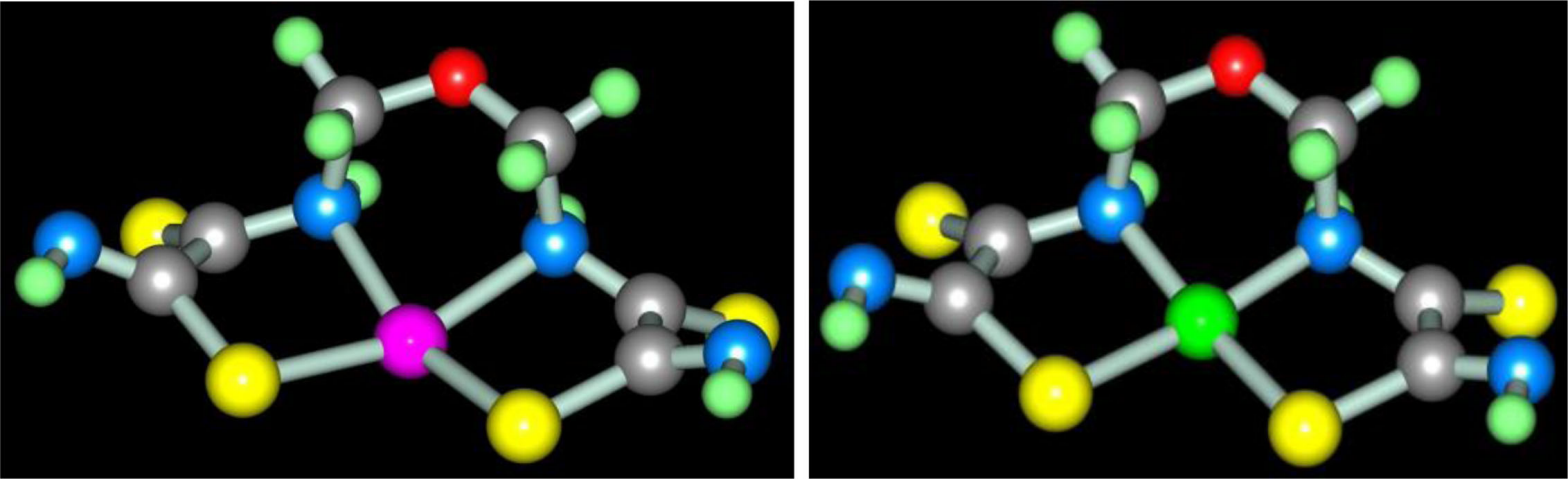
Molecular structures of [Co**L1]** (left) and [Ni**L1]** right). Here and further on.

In the works (46, 47), molecular structures of series of [M**L1]** metal complexes have been determined. The typical examples of molecular structures of such metal chelates are presented in Fig. 2.

As you can see, the structures of Co(II), Ni(II), and Cu(II) macrotricyclic complexes with 2,8-dithio-3,7-diaza-5-oxanonandithioamide-1,9 are extremely similar to each other. What puts of each of these complexes in a class by itself is that they, contrary to expectations, are non-coplanar; besides, both 5-numbered and 6-numbered chelate cycles having in their compositions, are non-coplanar, too. Among their number, the sum of valence angles in 6-numbered chelate cycle in the Co(II) complex is 620.8°, in the Ni(II) complex−629.8°, in the Cu(II) complex−623.0°; these values differ strongly from sum of inner angles in the plane hexagon (720°). As for metal chelate MN_2_S_2_ cycle, it is practically plane in the case of [Ni**L1]** and [Cu**L1]** complexes whereas in the case of [Co**L1]**, there are only donor atoms in one plane [sum of valence angles between metal atom and donor atoms (SMN), (NMN), (NMS) and (SMS) is 358.2° (Ni), 356.8° (Cu), 353.9° (Co); sum of non-valence angles (SNN), (NNS), (NSS) and (SSN) in the each of these complexes is 360.0°]. Note that dithiooxamide in the water-alkaline solution does not interact with formaldehyde, in any cases, at room temperature, therefore process (**3**.1) **can be** qualified as the template synthesis. In (43, 45), it has been postulated that it proceeds according to the following mechanism: at first, dithiooxamide is coordinated to Ni(II) via the donor N and S atoms, then formaldehyde comes into play as a ‘stitching agent’:


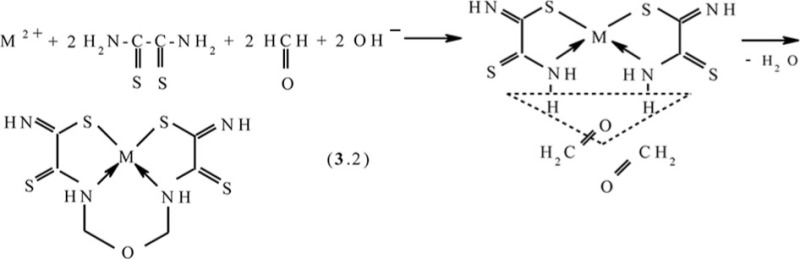


The fact that the ‘template’ complex obtained under scheme (**3**.1) is not formed at the contact between any Ni(II) chelate with dithiooxamide and formaldehyde either at the complex formation in **GIM** or at the complex formation in the solution or the solid phase (43) serves as an indirect confirmation in favor of such mechanism, A process similar to (**3**.1) is implemented at the complex formation in the Cu(II)–dithiooxamide–formaldehyde system into a Cu_2_[Fe(CN)_6_] **GIM** (41, 44, 48, 49). In the case of the Co(II)–dithiooxamide–formaldehyde system at the complex formation into a Co_2_[Fe(CN)_6_] **GIM**, the cobalt(II) complex formed according to the analogous reaction is only an intermediate and is immediately oxidized according to the reaction (**3**.3) into the heteroligand Co(III) complex containing the **L1** chelant along with H_2_O and the OH–group in the inner coordination sphere (44, 50, 51)


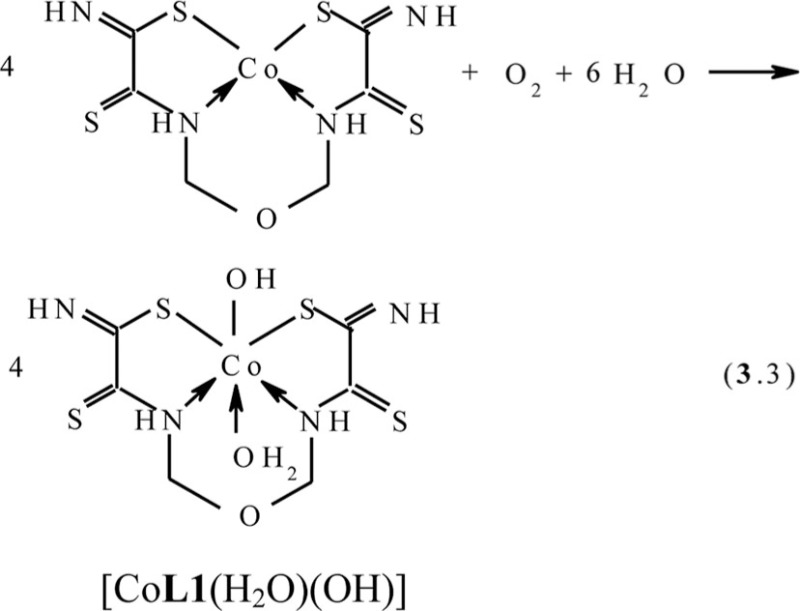


In the Co(III)–dithiooxamide–formaldehyde system at the complex formation into a KCo[Fe(CN)_6_] **GIM**, the coordination compound with the composition of [Co**L1**(H_2_O)(OH)] is formed in one stage according to the scheme (**3**.4) (52):


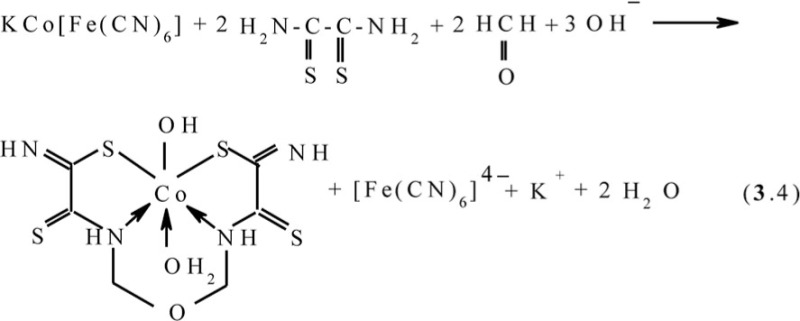


Processes analogous to (**3**.1) and (**3**.3) are implemented in the Fe(II)-dithiooxamide-formaldehyde system into a KFe[Fe(CN)_6_] **GIM** (53), however, they have yet to be studied in more detail.

**Fig. 3:**
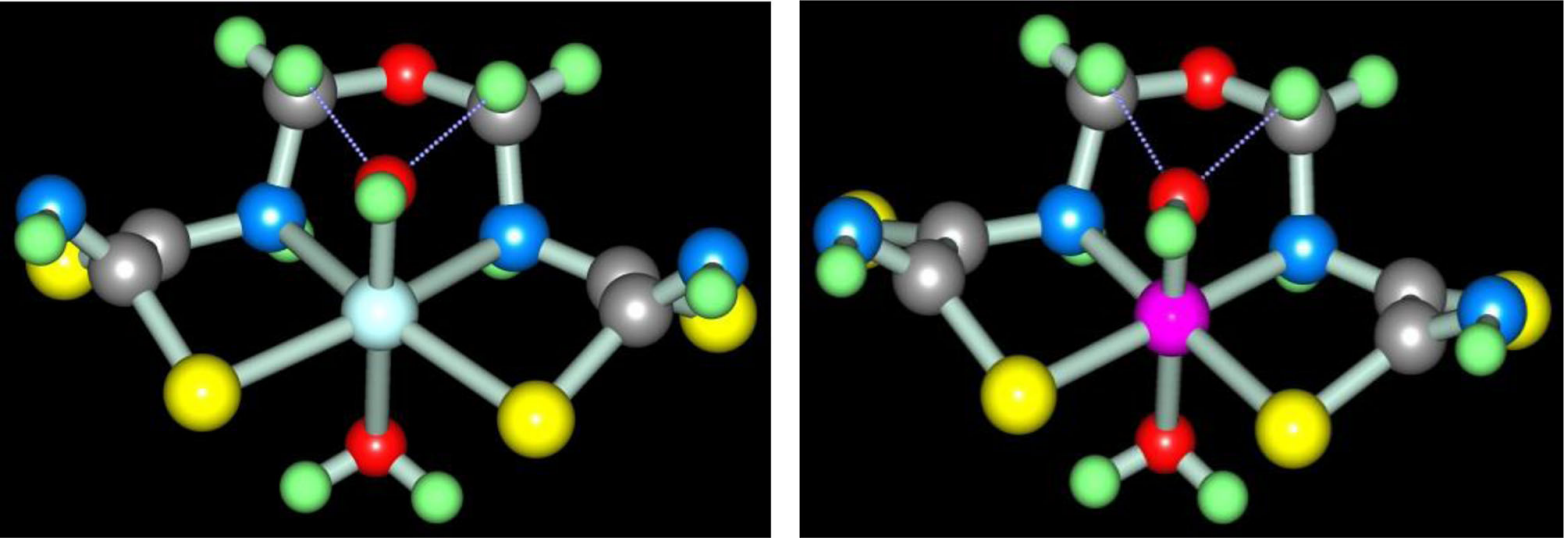
Molecular structures of [Fe**L1**(H_2_O)(OH)] (left) and [Co**L1**(H_2_O)(OH)] (right).

The molecular structures of the [M**L1**(H_2_O)(OH)] complexes (M=Fe, Co) were determined in (53, 54); they are shown in Fig. 3. To a certain extent, they resemble the structures of the [M**L1]** complexes, since additional six-numbered metal cycle formed as a result of template ‘cross-linking’, as in [M**L1]**, is not in the same plane with the MN_2_S_2_ grouping but, on the contrary, is very strongly declined [63.7° in the case of the Fe(III) complex and 55.5° in the case of Co(III)]. In this case, four atoms of six, contained in this cycle, namely two nitrogen and two carbon atoms, are in one plane; the plane (C)(O)(C) forms with (N)(C)(C)(N) plane the angle of 76.4° in the case of

[Fe**L1**(H_2_O)(OH)] and 75.8° in the case of [Co**L1**(H_2_O)(OH)]. For the Fe(III) complex as well as for the Co(III) complex, coplanar orientation of ligand **L1** donor atoms, relative to a corresponding metal ion, is energetically most favorable. In this case, the metal ion is located in one plane with **L1** donor atoms; the sum of angles (SMN), (NMS), (SMS) and (NMN) is very close to 360°. It should be noted that in both [M**L1**(H_2_O)(OH)] complexes indicated, the values of the above-mentioned angles are rather close to each other. As for M–O bonds with H_2_O and hydroxide-anion molecules, they, as should be expected *a priori*, are slightly different in their length from each other; in addition, the length of M–O bond for H_2_O [∼231.0 pm in the Fe(III) complex, ∼204.8 pm in the Co(III) complex] is more than the length of M–O bond for OH^−^ [∼183.8 pm in the Fe(III) complex, ∼182.9 pm in the Fe(III) complex].

In (45, 49, 54–59), the possibility of implementing the ‘soft’ template synthesis has been shown at the contact of MHF-**GIM** with the water-alkaline solutions containing dithiooxamide and such homologues of formaldehyde as acetaldehyde and acetone (M=Co, Ni, Cu). The kinetic curves and data of various physical and chemical methods indicate that, in the case of acetaldehyde, regardless of the template nature, the process proceeds according to the scheme (**3**.5) and is accompanied by the formation of the (**565**)macrotricyclic compounds with 4,6-dimethyl-2,8-dithio-3,7-diaza-5-oxanonandithioamide-1,9 **L2**


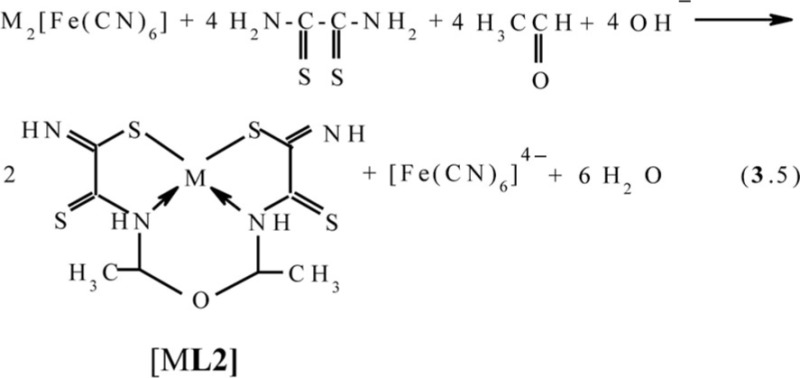


in the case of acetone, according to the scheme (**3**.6) with the formation of (**565**) compounds with 4,4,6-trimethyl-2,8-dithio-3,7-diazanonendithioamide-1,9 **L3**


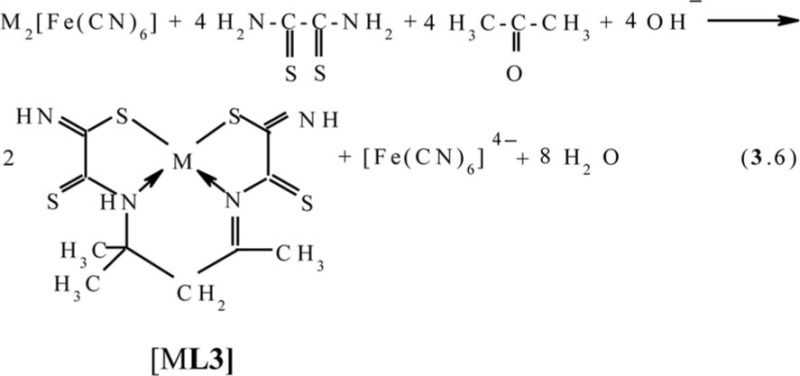


**Fig. 4:**
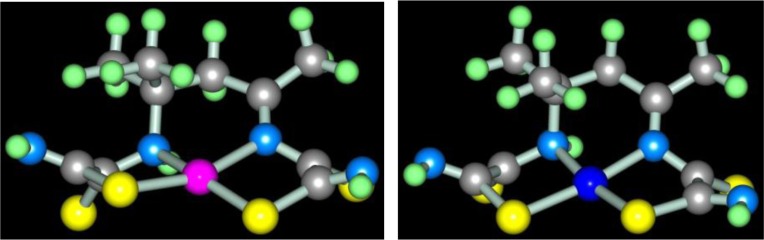
Molecular structures of [Co**L3]** (left) and [Cu**L3]** (right).

Molecular structures of complexes [M**L2]**, as it should be expected, are extremely similar to ones of corresponding complexes [M**L1]**. At the same time, molecular structures of [M**L3]** complexes, according to data (47, 60, 61), rather strongly differ from [M**L1]** ones; they are shown in Fig. 4. Note that, in the case of formaldehyde and acetaldehyde, the (N,S,S,N)-donor chelant formed as a result of self-assembly contains oxygen, whereas in the case of acetone it does not. In (45, 49), the possible mechanism of the reaction of the template synthesis in the M(II)–dithiooxamide–acetone system shown in the scheme (**3**.7) was proposed


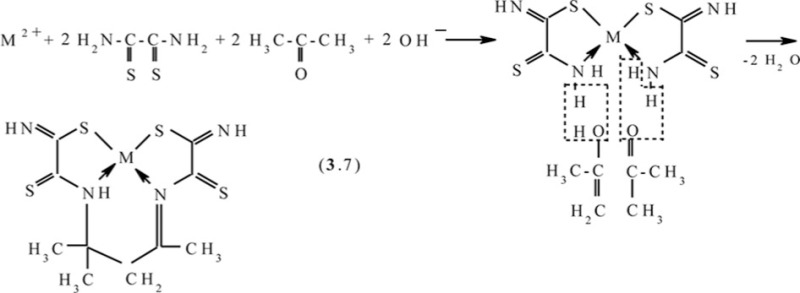


In KFe[Fe(CN)_6_] and KCo[Fe(CN)_6_] **GIM**, the processes of self-assembly (**3**.8) are implemented. On the one hand, they are reminiscent of the general processes of (**3**.4), and on the other hand, they are reminiscent of the general processes (**3**.6); therefore, similar [M**L3**(H_2_O)(OH)] (M=Fe, Co) complexes are formed (62, 63).


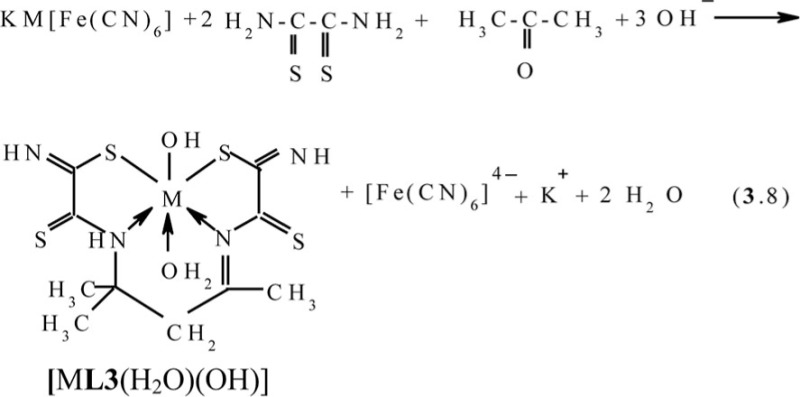


Rather recently in (64–69), the ‘soft’ self-assembly in the Cu(II)–dithiomalonamide–formaldehyde, Cu(II)–dithiomalonamide–acetone, and Cu(II)–thiocarbohydrazide–acetone systems was observed. According to the data of works cited above, in the first of these systems the process according to the scheme (**3**.9) with the formation of the Cu(II) (**666**) complex with 3,9-dithio-4,8-diaza-6-oxaundecadithioamide-1,11 **L4** occurs;


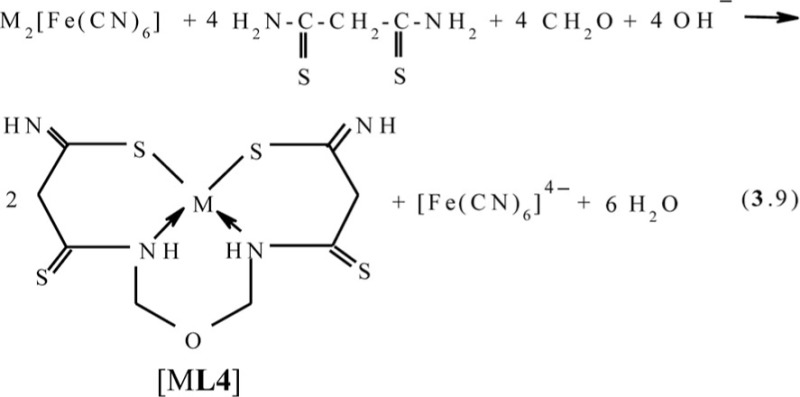


In the second system, the process according to the scheme (**3**.10) with the formation of the Cu(II) (**666**) complex with 5,5,7-trimethyl-3,9-dithio-4,8-diaza-undecene-7-dithioamide-1,11 **L5** occurs; and in the third system, with the formation of the heteroligand copper(II) (**565**) complex with 4,4,6-trimethyl-2,3,7,8-tetraazanonen-6-dithiohydrazide-1,9 **L6** according to the scheme (**3**.11), takes place.


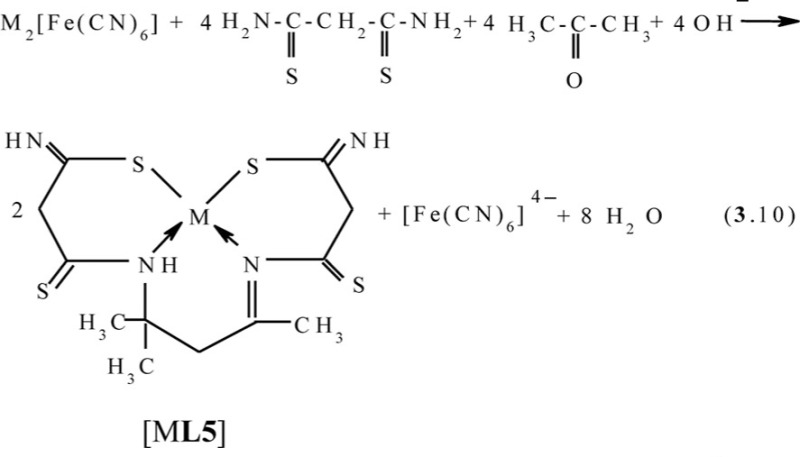



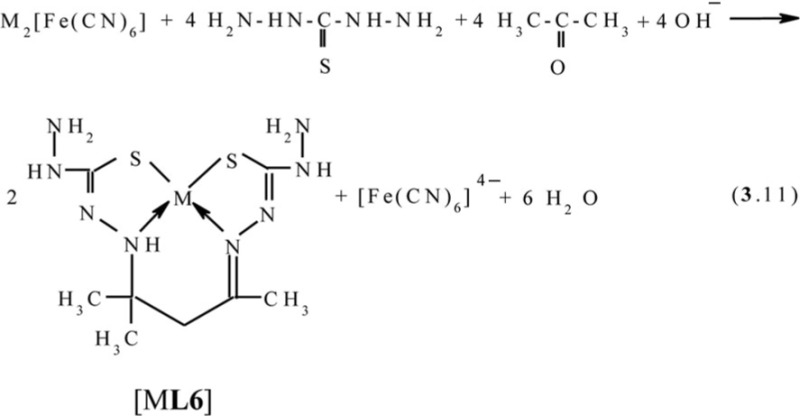


**Fig. 5:**
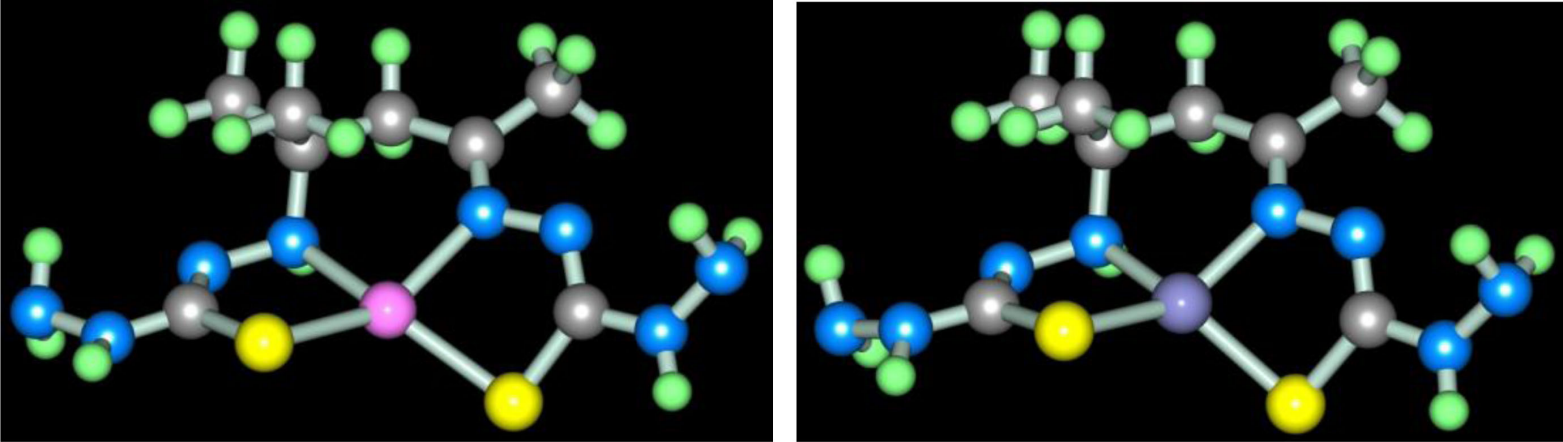
Molecular structures of [Mn**L6]** (left) and [Zn**L6]** (right).

Molecular structures of some [M**L6]** complexes were determined in (70, 71); two such structures are presented in Fig. 5. In full harmony with theoretical expectations, these complexes as well as the other above-mentioned (**565**)macrotricyclic compounds, are non-coplanar and have quasi-pyramidal orientation of N and S donor atoms to M(II). It should be noted in this connection that for Ni(II)–thiocarbohydrazide–acetone systems at the complexing in solutions, two other processes, namely (**3**.12) and (**3**.13), occur (M=Ni):


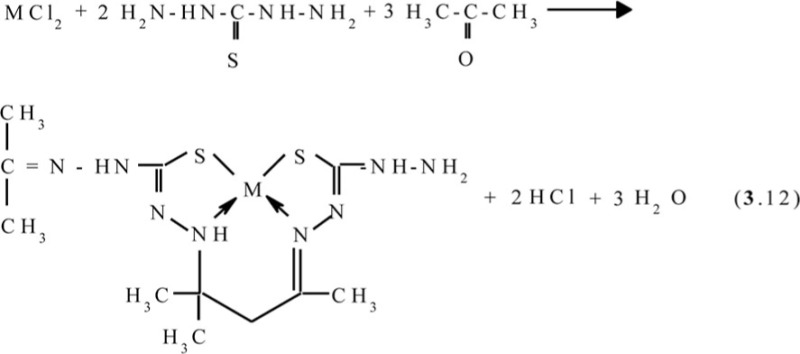



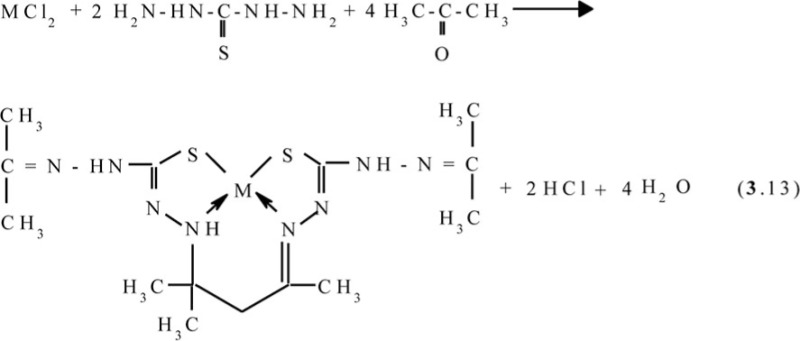


Note that, in the case of Cu(II), the processes of self-assembly are preceded by the alkaline destruction of Cu_2_[Fe(CN)_6_] to Cu(OH)_2_ hydroxide, which further reacts with the corresponding combination of ligsons. In the case of Ni(II), such a process does not occur and Ni_2_[Fe(CN)_6_] itself is apparently a stronger compound than the macrocyclic compounds which, in principle, could be formed in the above triple systems. Most likely, this is the reason that when copper(II) hexacyanoferrate (II) is replaced by the nickel(II) compound of a similar structure, no process of self-assembly (**3**.9–**3**.11) is implemented. When Co(II) plays the role of the template, the character of the proceeding processes is apparently somewhat different, but no detailed information on this subject is available in the literature (69).

**Fig. 6:**
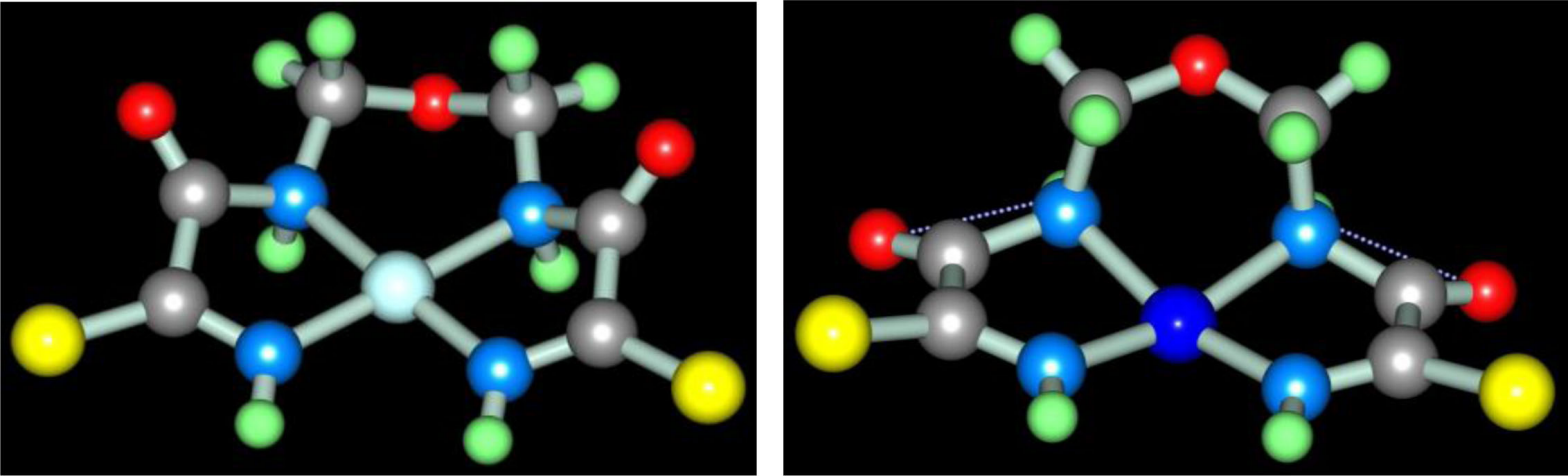
Molecular structures of [Fe**L7]** (left) and [Cu**L7]** (right).

In ref. (72), the opinion about possibility of self-assembly in the M(II)–thiooxamide–formaldehyde systems according to scheme (**3**.14) was voiced and molecular structures of a series 3*d*-elements [M**L7]** (**565**)macrotricyclic complexes formed as a result of this process were determined; some of them are presented in Fig. 6.


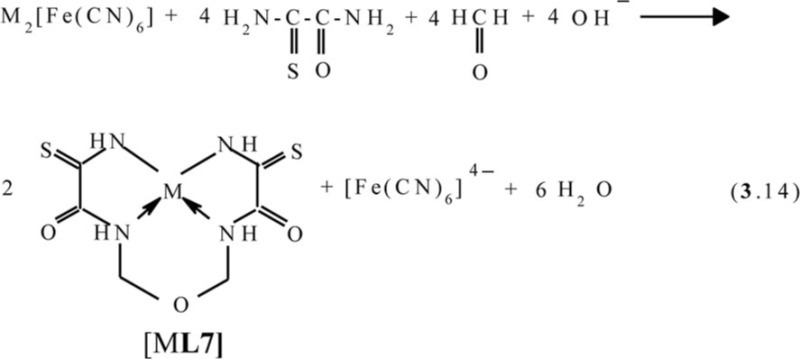


**Fig. 7:**
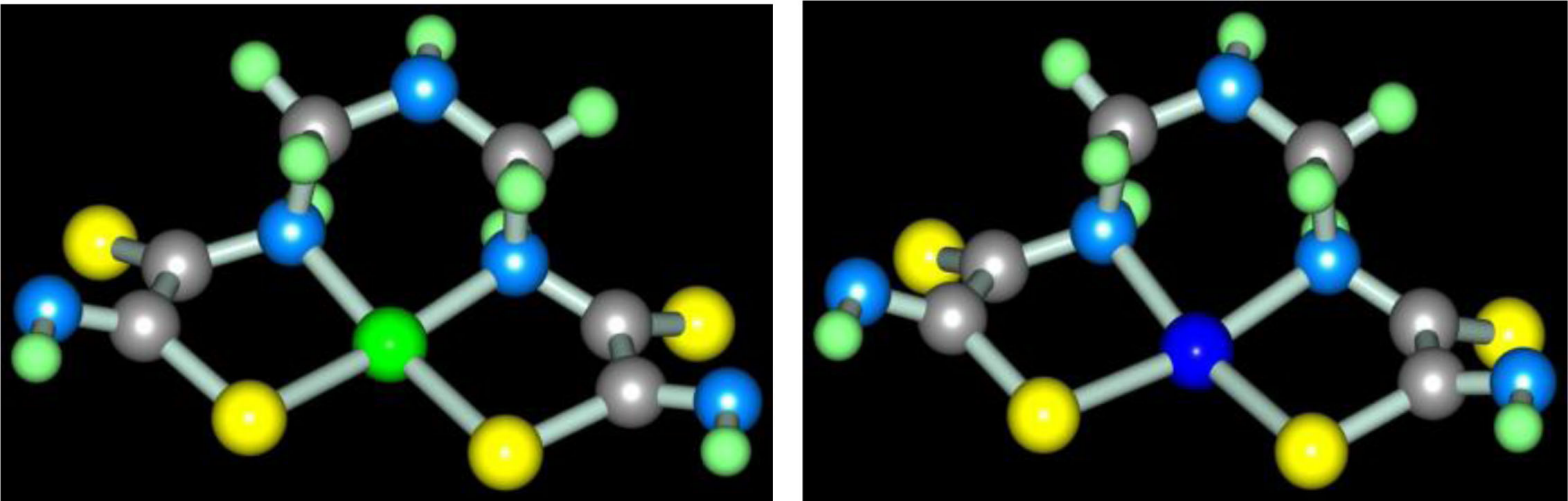
Molecular structures of [Ni**L8]** (left) and [Cu**L8]** (right).

According to ref. (73), self-assembly in the cobalt(II)-, nickel(II)-, and copper(II) hexacyanoferrate(II) **GIM** has been fixed at its contact with the water–alkaline solution containing three reagents: dithiooxamide, formaldehyde and ammonia. In a similar system, not triple- but four-fold, as each of these reagents performs the function of a ligson, the process of the complex formation proceeds according to the general equation (**3**.15) with the formation of the (**565**)macrotricyclic compound of M(II) ion with the 2,8-dithio-3,5,7-triazanonandithioamide-1,9 [M**L8]** where M=Co, Ni, Cu. The molecular structures of [M**L8]** complexes were considered in (74); as it was noted in the given article, these chelates are similar with [M**L1]** ones. These structures for Co(II), Ni(II), and Cu(II) complexes are shown in Fig. 7.


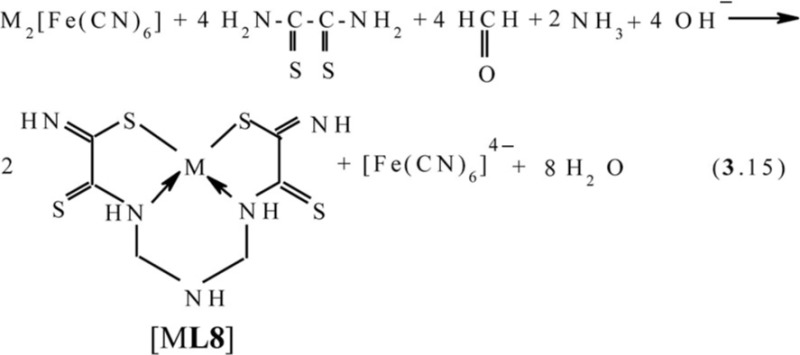


Molecular structures of these complexes, in whole, are like [M**L1]**, [M**L3]**, and [M**L7]** complexes and, also, are non-coplanar. In this connection, this non-coplanarity could be considered as general peculiarity of aza- and azathiatricyclic 3d-element complexes.

**Fig. 8:**
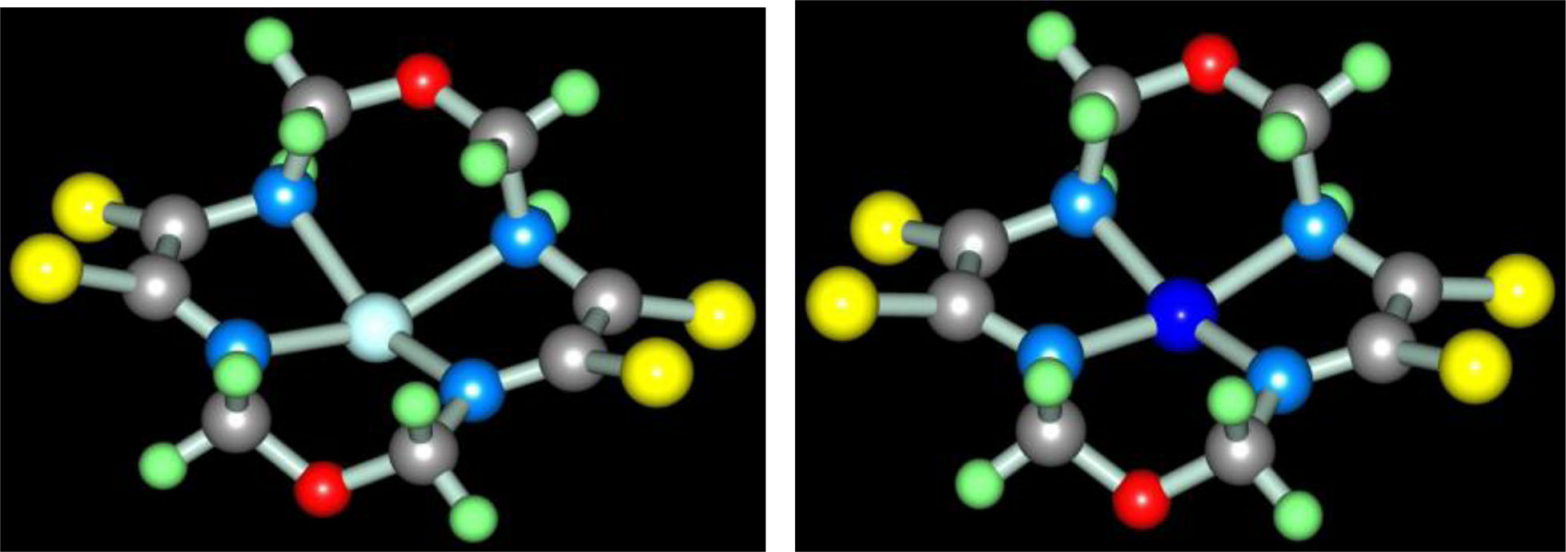
Molecular structures of [Fe**L9]** (left) and [Cu**L9]** (right).

**Fig. 9:**
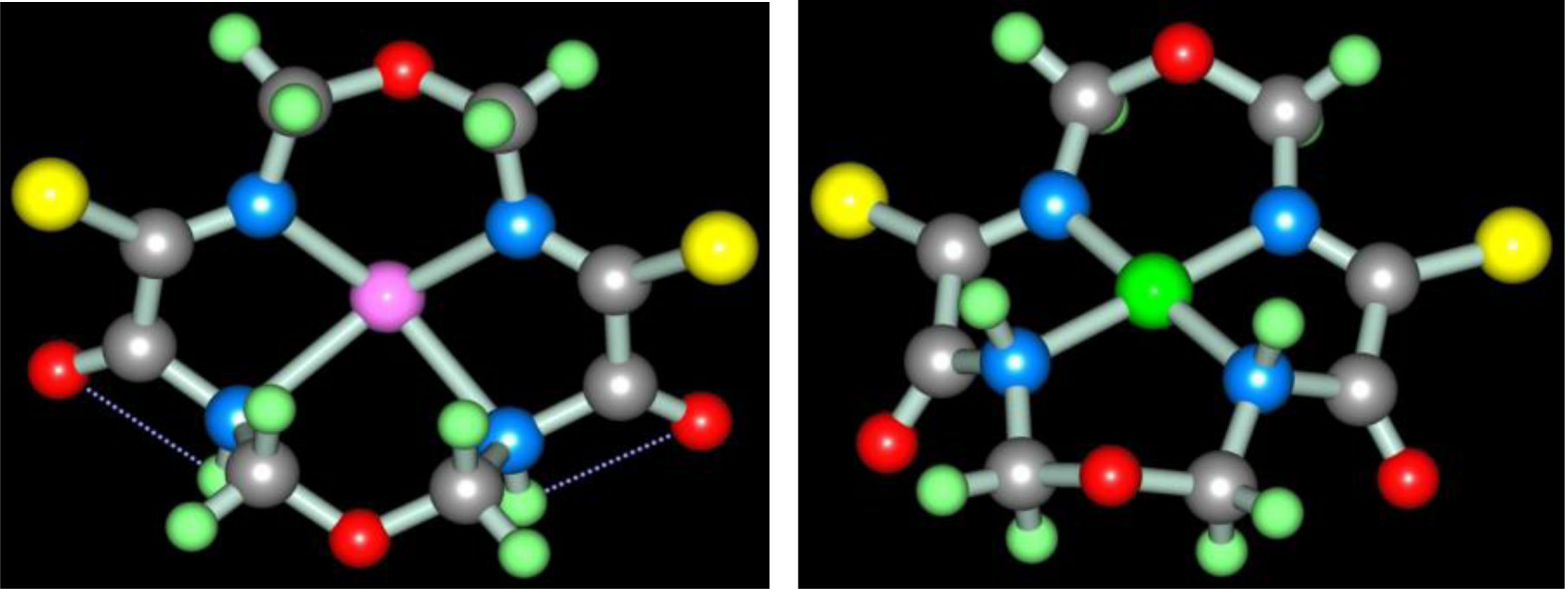
Molecular structures of [Mn**L10]** (left) and [Ni**L10]** (right).

**Fig. 10:**
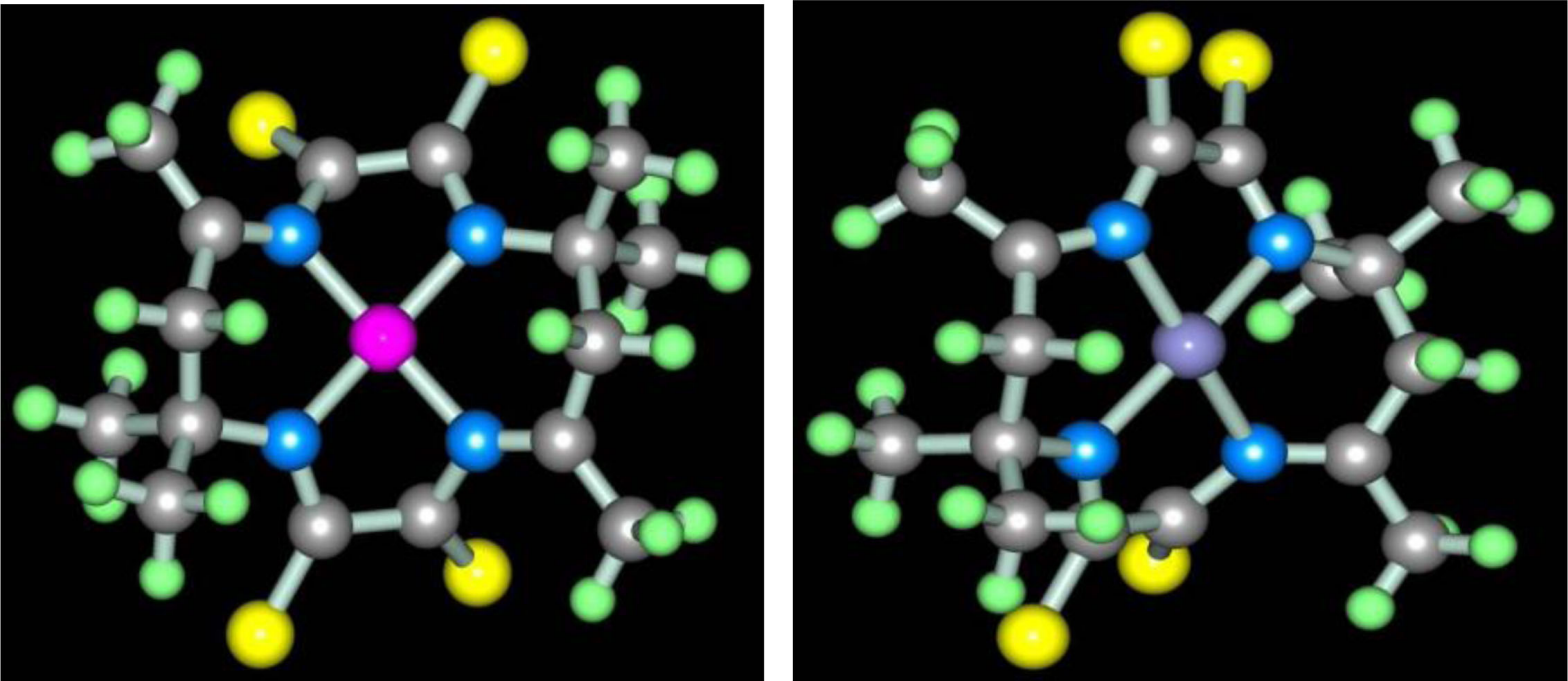
Molecular structures of [Co**L11]** (left) and [Zn**L11]** (right).

One can easily see that, in all of the above cases, as a result of self-assembly in MHF-**GIM**, the macrotricyclic metal complexes with two five-member- and one six-member cycles (**565**) or with three six-member cycles (**666**) are formed. There are some data that self-assembly with the formation of macrotetracyclic complexes of the (**5656**) type, for example, [M**L9]**, [M**L10]**, and **[**M**L11]**, according to the schemes (**3**.16–**3**.18), is possible (75–77). Molecular structures of some of them are shown in Figs. 8–10.


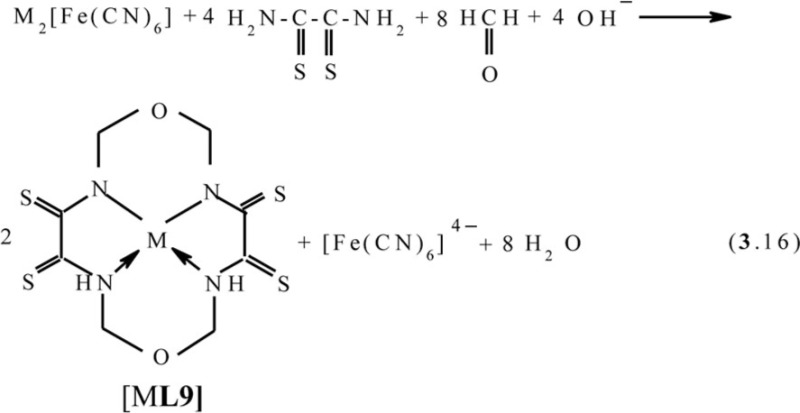



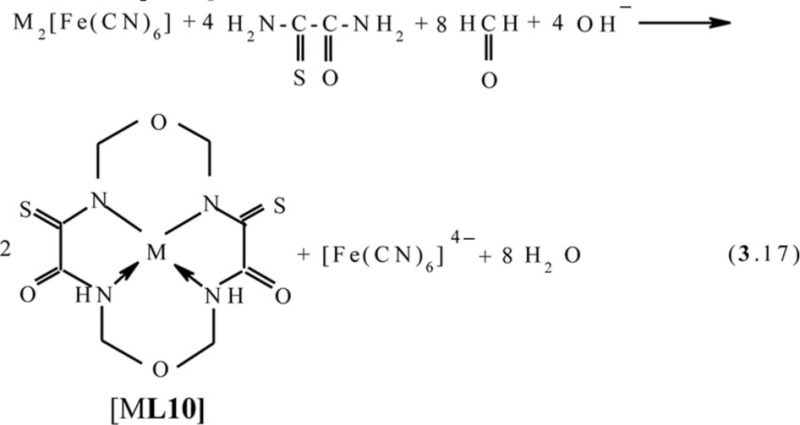



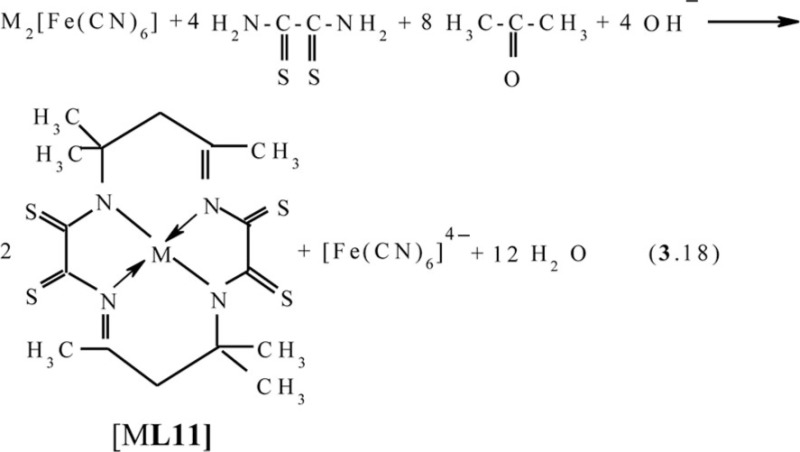


It should be noted in this connection that none of these macrotetracyclic complexes are plane; moreover, none of the 5-numbered and 6-numbered cycles, are plane either (75–77).

## 3. Self-assembly of aza- and azathiamacrocyclic metal chelates with the participation of (N,S)-ligsones and dicarbonyl compounds

It is easy to notice that, when monocarbonyl compounds are used for ‘stitching’ the chelate cycles by the (N,S) ligson A in a single cyclic contour, at least two molecules of ligson B per one 3*d*-element atom are required. Thus, a certain structural reorganization occurs and, as a result, in some cases, namely in the cases of formaldehyde and acetaldehyde, an oxygen atom appears in the additional chelate cycles; in other cases, namely in the case of acetone, this cycle contains only carbon and nitrogen atoms. Interestingly, this additional cycle, as a rule, contains six atoms. The usage of dicarbonyl compounds in which the carbon atoms forming the C=O groups are close or separated by one, two, or three atoms, in the processes of self-assembly allows the implementation of one more possibility of ‘stitching’ supposed theoretically when the additional cycle is formed at the participation of only one molecule of the carbonyl-containing ligson per one metal atom; in such a variant, this cycle can be both six-member and with another number of atoms, for example, 5, 7, or 8.

The simplest dicarbonyl compound is known to be glyoxal HC(O)–CH(O). This compound has been used as a ‘stitching’ ligson in combination with the above-mentioned dithiooxamide in (43, 51, 66–72). In this case, in Ni_2_[Fe(CN)_6_]- and Cu_2_[Fe(CN)_6_]-**GIM**, the process proceeds according to the generalized scheme (**4**.1)


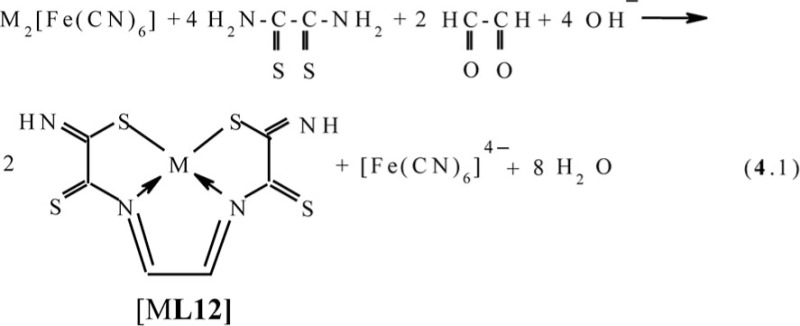


References (43, 78–80) and (78, 79, 81, 84), respectively, lead to the formation of the (**555**) macrotricyclic metal chelates with the (NSSN)-donor tetradentate chelant, 2,7-dithio-3,6-diazaoctadien-3,5-dithioamide-1,8 [M**L12]**, where M=Ni, Cu. In (43), the mechanism of this process described by scheme (**4**.2) was proposed. In the case of Co_2_[Fe(CN)_6_]-GIM, a self-assembly process proceeds in two stages; first is the reaction analogous to (**4**.1), and second is the oxidation of Co(II) to Co(III) according to the scheme (**4**.3) with formation of [M**L12**(H_2_O)(OH)] macrotricyclic chelate where M= Co (44, 49, 82)


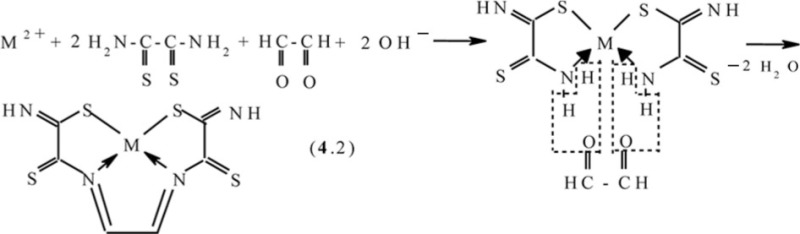



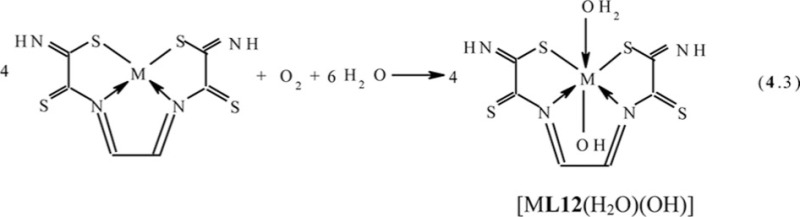


In KCo[Fe(CN)_6_]-**GIM**, the process of self-assembly proceeds according to the scheme (**4**.4) and is accompanied by the formation of the same [Co**L12**(H_2_O)(OH)] macrotricyclic compound as in the Co(II)–dithiooxamide–glyoxal system (44, 51, 72, 73) (M=Co)


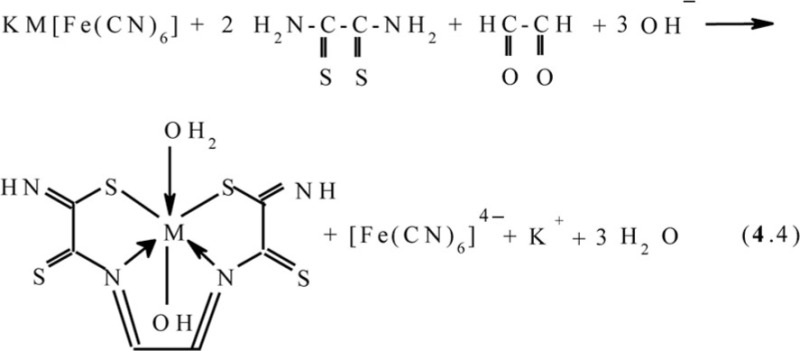


**Fig. 11:**
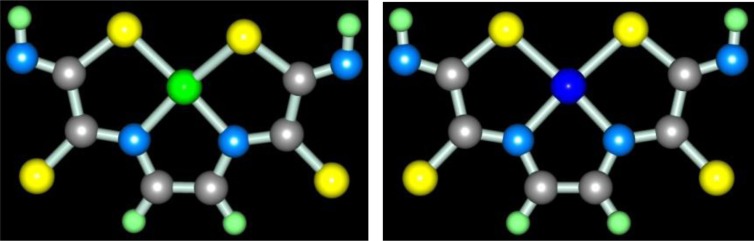
Molecular structures of [Ni**L12]** (left) and [Cu**L12]** (right).

[M**L12]** molecular structures were considered in (86); for example, structures of Ni**L12** and Cu**L12** are shown in Fig. 11.

One can easily note that this situation resembles the above situation in the Co(II) (Co(III))–dithiooxamide–formaldehyde systems.

Interestingly, in spite of the extensive literature on the processes of self-assembly of the metal macrocyclic compounds (for example, in (7, 9) more than 1,000 references to the publications on the given problem are given), no possibility of self-assembly even in one of the above systems in the solution or the solid phase is mentioned. Moreover, dithiooxamide, in spite of the availability of four mobile hydrogen atoms, has been never used as a ligson for template synthesis up until now. Apparently, this is due to the rather low proton donor ability of both dithiooxamide and its coordination compounds (and, respectively, the low mobility of protons at the nitrogen atoms coordinated to the metal ions) which is considerably enhanced in the gelatin bulk in the alkaline medium where the molecules of the given polymer, as was mentioned above, get the negative charge.

Recently, the ‘soft’ template synthesis in the Cu(II)–dithiomalonamide–diacetyl (87, 88) and Cu(II)–thiocarbohydrazide–diacetyl (89) systems was observed. According to the published data, in the first of these systems, the process (**4**.5) with the formation of the macrotricyclic complexes of the (**656**) type with the (NSSN) donor tetradentate chelant, 5,6-dimethyl-1,10-diamine-1,10-dimercapto-4,7-diazadecatetraen-1,4,6,9-dithione-3,8 [M**L13]** (M=Ni, Cu) takes place


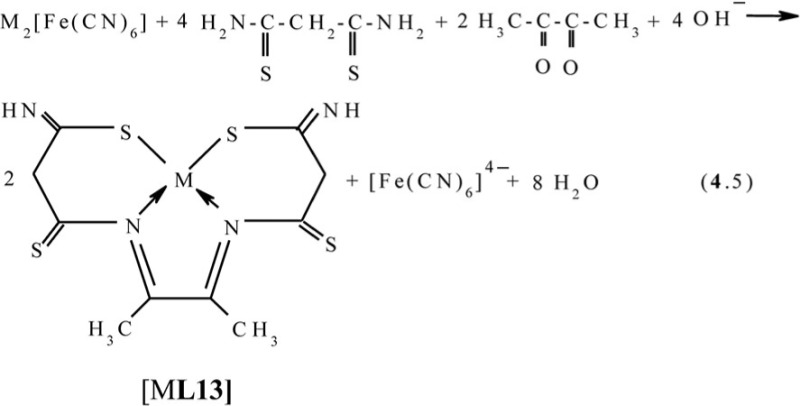


In the second of these systems, the formation of three complexes with two various chelants, namely two (**656**)macrotricyclic ones with 4,5-dimethyl-2,3,6,7-tetraazaoctadien-3,5-dithiohydrazide-1,8 **L14** and one (**5656**)macrotetracyclic ones with 3,10-dithio-6,7,13,14-tetramethyl-1,2,4,5,8,9,11,12-octacyclotetradecatetraene-1,5,7,12 **L15**, according to the general scheme (**4**.6) (89), is observed (M= Cu)


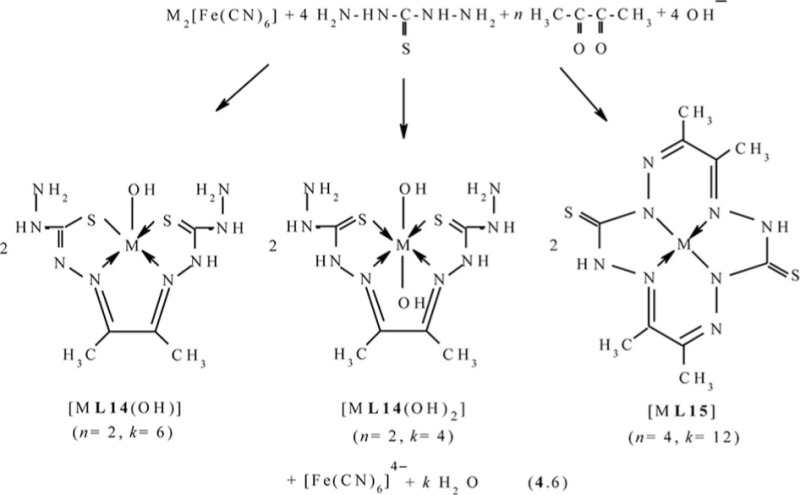


**Fig. 12:**
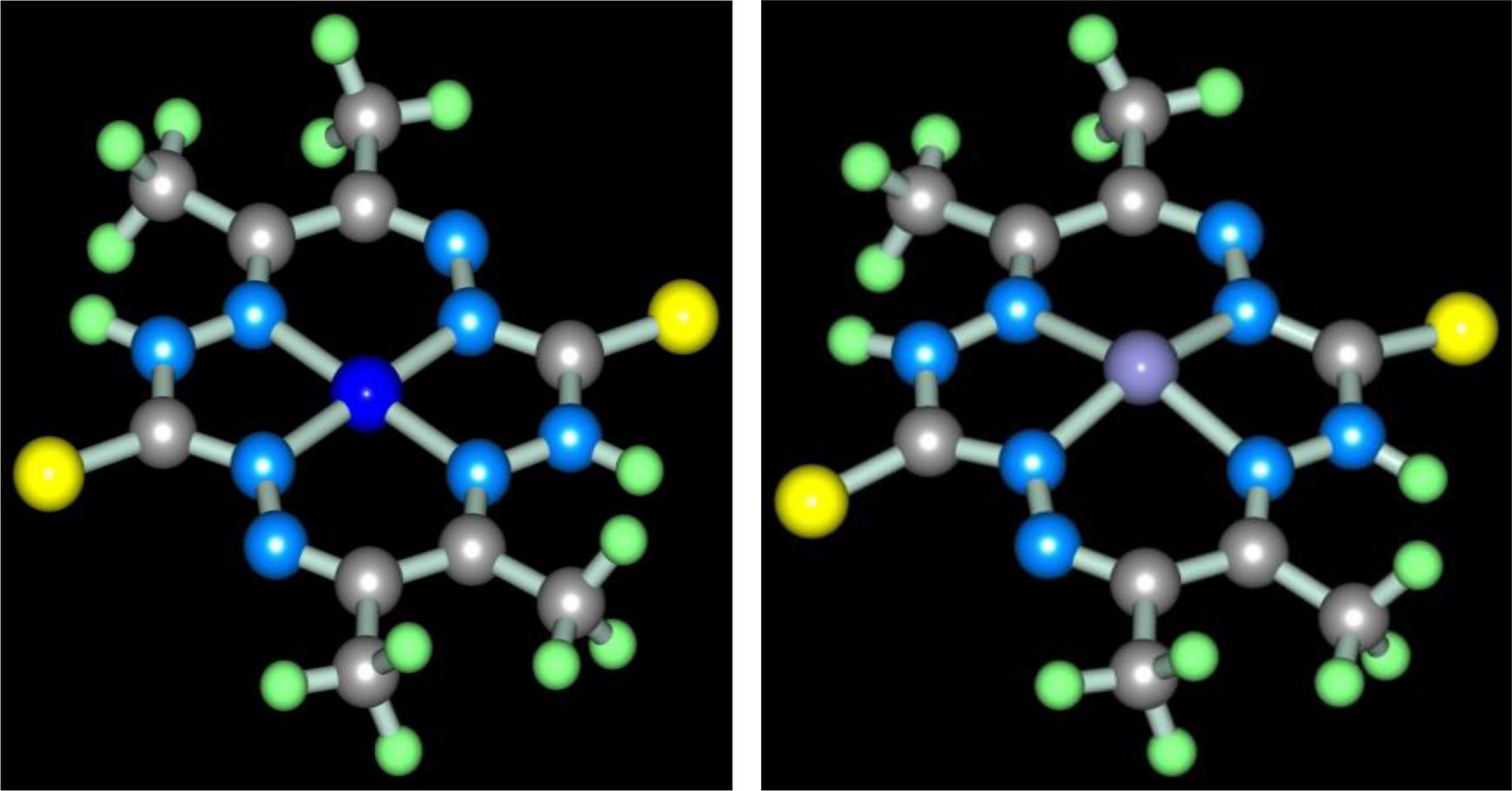
Molecular structures of [Cu**L15]** (left) and [Zn**L15]** (right).

Molecular structure of some [M**L15]** complexes was studied in (90); some of them are presented in Fig. 12. Because four non-planar methyl groups are the constituents of the test chelates, note that none of them can possess a fully coplanar structure. Nevertheless, if we mentally remove these groups from the examination and take into consideration only chelate units and metal chelate rings for evaluating the structure coplanarity, we find that the complex of Cu(II) is ideally planar; the complexes of Fe(II), Co(II), and Ni(II) are almost ideally planar; and the complexes of Mn(II) and Zn(II) are non-coplanar only. Thus, a number of such complexes have a perfect or near perfect flat 14-membered macrocycle, which in itself is quite remarkable (if only because, even 8-membered cycles, as it is well-known in organic chemistry, are rarely flat).

Apart from glyoxal and diacetyl, as dicarbonyl ligson in the processes of self-assembly in the MHF-**GIM** has now tested only acetylacetone; this ligson was described in (91), where the process (**4**.7) with the formation of (**565**)macrotricyclic complexes [M**L16]** was realized


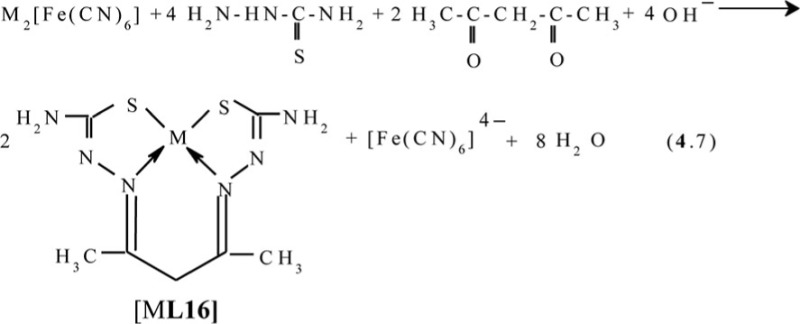


**Fig. 13:**
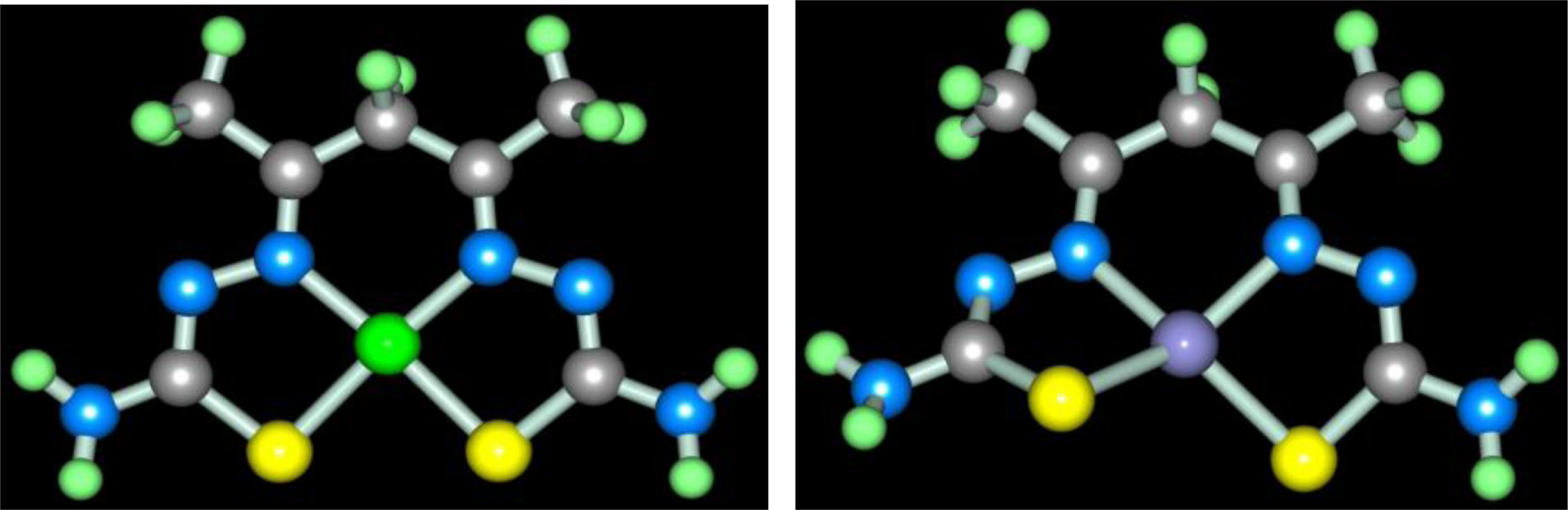
Molecular structures of [Ni**L16]** (left) and [Zn**L16]** (right).

In (92) the molecular structure of these complexes has been described in detail; some of them, namely the structure of the Ni(II) and Zn(II) chelates, are shown in Fig. 13. A very interesting feature of complexes considered in (92), is that available in each of the 6-membered metal chelate cycles are essentially plane (sum of bond angles in them is different from the sum of the internal angles of the plane hexagon not more than 0.5°), although overall, they, like others previously considered (565)macrotricyclic metal chelates with N and S atoms in the macrocycle, are non-coplanar.

It should be noted that the processes of self-assembly are implemented in a number of other triple systems containing dithiooxamide, dithiomalonamide, and thiocarbohydrazide as a (N,S)-ligson; however, the composition and the molecular structure of the metal complexes formed and chelants in their internal coordination sphere has to be specified.

At the present time, there are a lot of publications in literature where self-assembly processes of azamacrocyclic and thiaazamacrocyclic chelates were described (see, e.g, (7, 9, 93–110); in this connection, there are strong reasons to believe that the possibilities of the template synthesis in MHF **GIM** are high enough and the above list of triple metal ion–ligand synthon A–ligand synthon B systems in which it is implemented can be considerably expanded.

## 4. Nanoparticles formed as a result of molecular nanotechnologies of self-assembly in MHF GIM

**Fig. 14:**
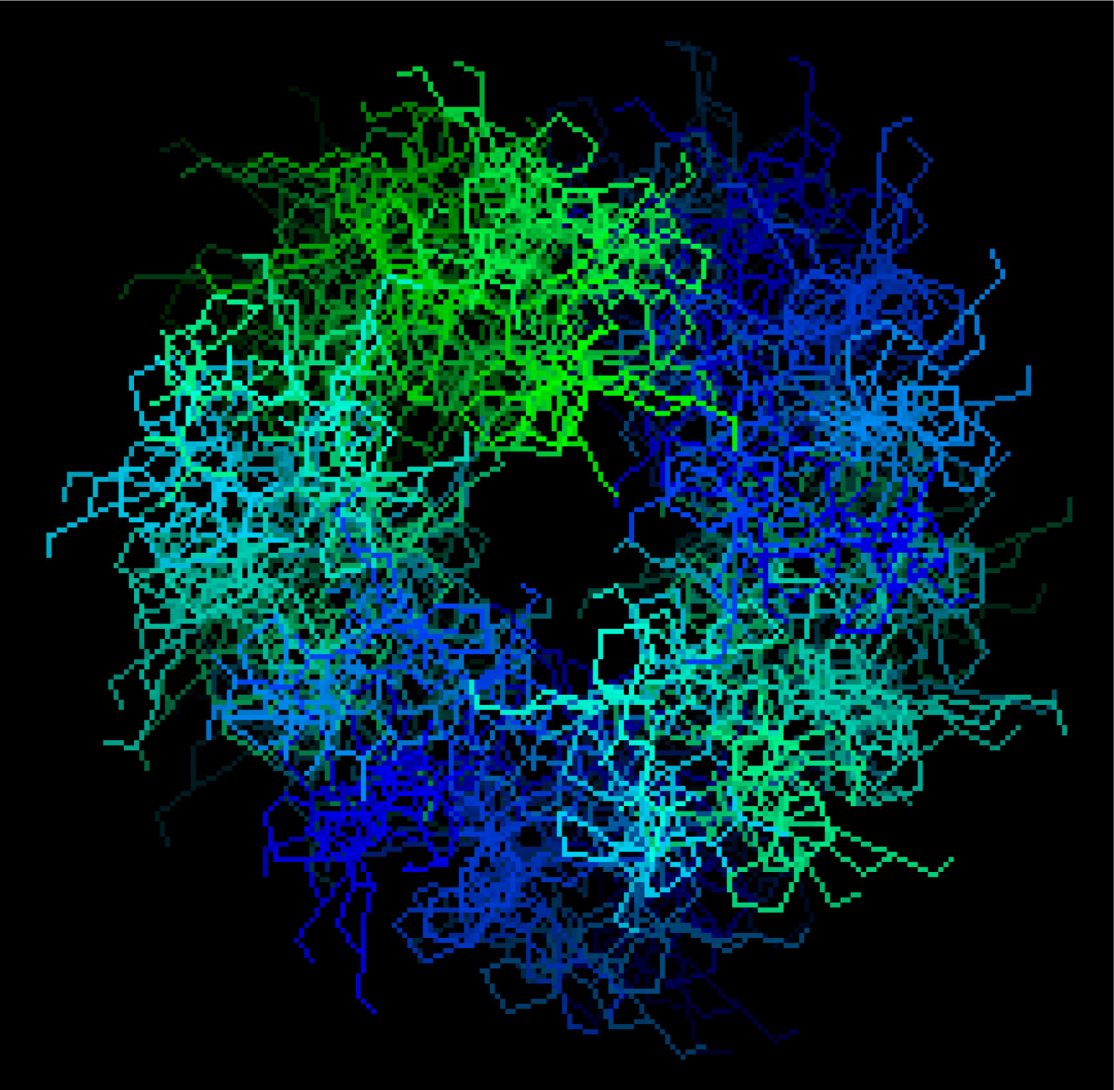
The general plane of intermolecular cells in which can be *nano*-particles of MHF and macrocyclic metal chelates formed in a self-assembly process in the MHF **GIM**.

Potentially, the gelatin structure is rather convenient for the formation of the immobilized matrix systems, since, on the one hand, it does not allow any rigid crystal blocks to be implemented and, on the other hand, it has enough cells to take and fix molecules of the immobilized chemical compound; in addition, these cells, even being filled with such molecules, keep some freedom of motion in the space. The general plane of gelatin mass with such intermolecular cells is presented in Fig. 14.

One can try to estimate the size of such a cell. In fact, the volume of a polymeric gelatinous layer (*V*_Gel_) with the area of 1 cm^2^ and 20 µm thick is (1.0•1.0•20•10^−4^) cm^3^=2.0•10^−3^ cm^3^, so the weight of gelatin it contains at its average density value of 0.5 g/cm^3^ is (0.5•2.0•10^−3^) g=1.0•10^−3^ g. Since, as mentioned above, the molecular mass of gelatin (*M*_Gel_) is about (2.0–3.0) •10^5^, the number of its molecules in the mass of 1.0•10^−3^ g, is (1.0•10^−3^/*M*_Gel_)•6.02•10^23^)=(2.0–3.0)•10^15^. It was already noted above that the gelatin molecule on average has a length of 2,850 nm and a width of 14 nm; if it is assumed that the gelatin molecule can be presented as a narrow-band cylinder, its volume *V*_*M*_ is (1/4)π*D* ^2^ *h=*(1/4)•3.14•(2,850•10^−8^ cm)•(14•10^−8^ cm)^2^)=4.38•10^−19^ cm^3^. Upon maximally dense packing, these molecules take the total volume of (4.38•10^−19^ • (2.0–3.0) •10^15^)=(8.76–13.15)•10^−4^ cm^3^. It is possible to postulate that the volume of cells-voids of interest is the total volume of the polymeric bulk minus the volume just calculated above taken by the gelatin molecules [2.0•10^−3^ – (8.76–13.15)•10^−4^] cm^3^, being (0.69–1.12)•10^−3^ cm^3^. Then, the average volume of one intermolecular hollow can be found as a quotient from division of their total volume into the number of gelatin molecules and, as it can be easily noted, will be (0.69–1.12)•10^−3^ cm^3^: (2.0–3.0)•10^15^=(2.3–5.6)•10^−19^ cm^3^=(2.3–5.6)•10^2^ nm^3^. The *linear* size of such an ‘average’ cell assuming its spherical form is *D=*(6*V*/π)^1/3^=[6•(2.3–5.6)•10^−19^ cm^3^/3.14)]^1/3^=(**7.60–10.22**) nm; for the cubic form it is *a=V* ^1/3^=[(2.3–5.6)•10^−19^ cm^3^]^1/3^=(**6.13–8.24**) nm. One can see from these values that, gelatin mass contains the multitude of *nano*-sized cells; on the other hand, at similar sizes of a cell, it is possible to introduce large enough molecules of the immobilized substance in it. It should be noted in this connection that, according to our data, MHF **GIM**, in whole, are transparent to electron flow and contain *nano*-particles with sizes <100 nm. Thus, it may be believed that any such **GIM** has the *nano* level of structure organization and, hence, may belong to systems with a preliminary decrease in entropy. On the other hand, by taking into consideration the intermolecular cell ‘average’ size which was indicated above (∼7–11 nm), it may be expected that the particles of gelatin-immobilized macrotricyclic and macrotetracyclic coordination compounds formed in these *nano*-sized intermolecular cells, indeed, will have linear sizes no more than 100 nm.

**Fig. 15:**
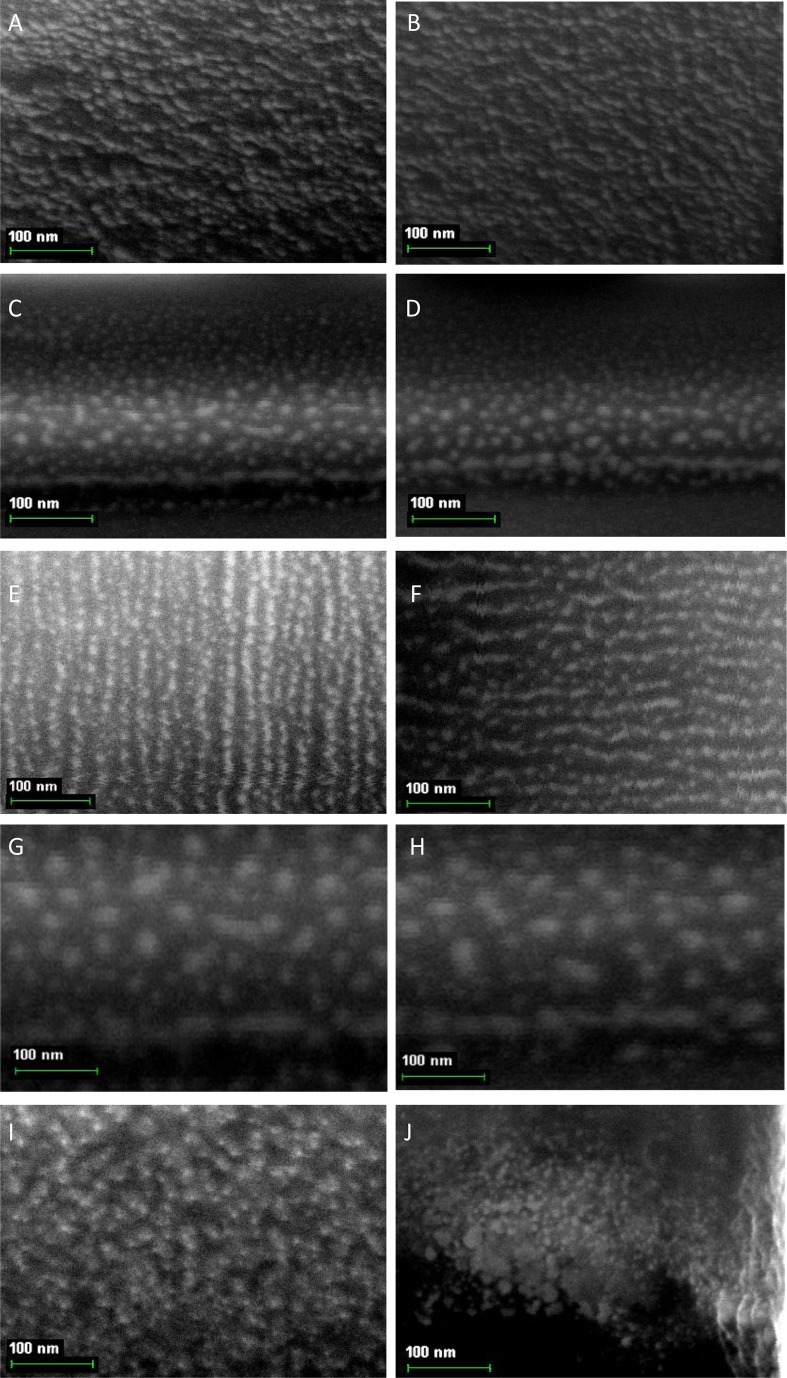
The SEM photos of *nano*-particles of macrocyclic metal chelate formed in a self-assembly process in the MHF **GIM** in the Co(II)–dithiooxamide–formaldehyde (A), Co(II)–dithiooxamide–glyoxal (B), Ni(II)–dithiooxamide–formaldehyde (C), Ni(II)–dithiooxamide–glyoxal (D), Ni(II)–thiocarbohydrazide–formaldehyde (E), Ni(II)–thiocarbohydrazide–acetone (F), Cu(II)–dithiooxamide–formaldehyde (G), Cu(II)–dithiooxamide–glyoxal (H), Cu(II)–thiocarbohydrazide–formaldehyde (I) and Cu(II)–thiocarbohydrazide–diacetyl (J).

In Fig. 15, the photos of *nano*-particles of macrocyclic metal chelates, arising in the MHF-**GIM** at self-assembly processes in some of triple systems considered, namely Co(II)–dithiooxamide–formaldehyde, Co(II)–dithiooxamide–glyoxal, Ni(II)–dithiooxamide–formaldehyde, Ni(II)–dithiooxamide–glyoxal, Ni(II)–thiocarbohydrazide–formaldehyde, Ni(II)–thiocarbohydrazide–acetone, Cu(II)–dithiooxamide–formaldehyde, Cu(II)–dithiooxamide–glyoxal, Cu(II)–thiocarbohydrazide–formaldehyde and Cu(II)–thiocarbohydrazide–diacetyl, are presented. As can be seen from them, the sizes of *nano*-particles formed under specific conditions, are in the 20–40 nm range. It should be noted in this connection that the shape of these *nano*-particles, however, has not been clearly defined and no correlation between this shape and nature of complexes formed in these systems, is observed.

## 5. Conclusion

The information available at present, fragments of which are presented in this review, allows one to state that one more ‘branch’ in molecular nanotechnology, self-assembly, of metal macrocyclic compounds in the biopolymer-immobilized matrix systems at the rigid fixation of a metal ion and the relatively high mobility of the organic compounds participating in self-assembly is being established. This field of nanochemistry operates with its own objects and is based on constructive principles and methodical and methodological approaches. The present stage of its development, however, is rather far from its apogee regarding both the accumulation of the experimental data and their theoretical understanding, generalization, and systematization. Anyway, at the present stage of the development of this field of molecular nanotechnology, a lot of problems have arisen and it would be good to mention some of them.

First of all, this includes improving the model for a description of the kinetics of self-assembly in MHF **GIM**. One ‘sketch’ of such a model has been given by the author of this review in article (111). However, this model and its mathematical apparatus do not consider the nature of the metal ion M in MHF, let alone the nature of ligsons interacting with it. In addition, these reagents are actually considered particles with sizes extremely small if compared with the sizes of the pores of the polymeric bulk in which they diffuse during self-assembly. Neither the first nor the second approximation can be considered completely correct (though on the whole this model yields quite good results when applied in practice). Another problem is to reveal the role of the gelatin molecules in the ‘choice’ of the actual mechanism of the self-assembly processes happening in **GIM** as an organizing medium.

The problem with the considerable expansion of the variety of metal complex **GIM** used in the processes of self-assembly is one of the fundamental pragmatic problems. There are no obstacles in this respect from the theoretical point of view (except for the substance immobilized in **GIM** not having a low enough solubility in water). From our point of view, in the near future, self-assembly with the participation of the gelatin-immobilized heteronuclear and cluster metal complexes, in particular heterobinuclear and heteropolynuclear MHF various ions of the *p-*, *d-*, and *f-*elements, may get a considerable impetus for development. As to the problems of the particularly pragmatic aspect, revealing the possibilities of the practical use of **GIM** containing metal macrocyclic compounds formed in the processes of self-assembly is undoubtedly of highest priority. Thus, it is rather probable that such systems will be effective catalysts of a number of practically important processes both in ‘conventional’ chemical technology and in nanotechnology. Since gelatin refers to the category of the hydrophilic biopolymers, it is doubtless that similar **GIM** in the future can find their niche in biochemistry and biophysics, and medicine and pharmacology. Revealing the particular possibilities of the gelatin-immobilized metal macrocyclic compounds could be considered one of the current problems of modern molecular nanotechnology.
